# Identifying treatment heterogeneity in atrial fibrillation using a novel causal machine learning method

**DOI:** 10.1016/j.ahj.2023.02.015

**Published:** 2023-03-07

**Authors:** Che Ngufor, Xiaoxi Yao, Jonathan W. Inselman, Joseph S. Ross, Sanket S. Dhruva, David J. Graham, Joo-Yeon Lee, Konstantinos C. Siontis, Nihar R. Desai, Eric Polley, Nilay D. Shah, Peter A. Noseworthy, Rochester, MN; New Haven, CT; San Francisco, CA; Silver Spring, MD; Atlanta, GA; Chicago, IL

**Affiliations:** a Robert D. and Patricia E. Kern Center for the Science of Health Care Delivery, Mayo Clinic, Rochester, MN,; b Department of Artificial Intelligence and Informatics, Mayo Clinic, Rochester, MN,; c Division of Health Care Delivery Research, Mayo Clinic, Rochester, MN,; d Department of Internal Medicine, Section of General Internal Medicine, Yale School of Medicine, New Haven, CT,; e Center for Outcomes Research and Evaluation, Yale-New Haven Hospital, New Haven, CT,; f Department of Medicine, University of California, San Francisco School of Medicine, San Francisco, CA,; g Section of Cardiology, Department of Medicine, San Francisco Veterans Affairs Medical Center, San Francisco, CA,; h Office of Surveillance and Epidemiology, Center for Drug Evaluation and Research, U.S. Food and Drug Administration, Silver Spring, MD,; i Office of Bio-statistics, Center for Drug Evaluation and Research, U.S. Food and Drug Administration, Silver Spring, MD,; j Department of Cardiovascular Medicine, Mayo Clinic, Rochester, MN,; k Delta Airlines, Atlanta, GA,; l Department of Public Health Sciences, University of Chicago, Chicago, IL

## Abstract

**Background:**

Lifelong oral anticoagulation is recommended in patients with atrial fibrillation (AF) to prevent stroke. Over the last decade, multiple new oral anticoagulants (OACs) have expanded the number of treatment options for these patients. While population-level effectiveness of OACs has been compared, it is unclear if there is variability in benefit and risk across patient subgroups.

**Methods:**

We analyzed claims and medical data for 34,569 patients who initiated a nonvitamin K antagonist oral anticoagulant (non-vitamin K antagonist oral anticoagulant (NOAC); apixaban, dabigatran, and rivaroxaban) or warfarin for nonvalvular AF between 08/01/2010 and 11/29/2017 from the OptumLabs Data Warehouse. A machine learning (ML) method was applied to match different OAC groups on several baseline variables including, age, sex, race, renal function, and CHA_2_DS_2_ -VASC score. A causal ML method was then used to discover patient subgroups characterizing the head-to-head treatment effects of the OACs on a primary composite outcome of ischemic stroke, intracranial hemorrhage, and all-cause mortality.

**Results:**

The mean age, number of females and white race in the entire cohort of 34,569 patients were 71.2 (SD, 10.7) years, 14,916 (43.1%), and 25,051 (72.5%) respectively. During a mean follow-up of 8.3 (SD, 9.0) months, 2,110 (6.1%) of patients experienced the composite outcome, of whom 1,675 (4.8%) died. The causal ML method identified 5 subgroups with variables favoring apixaban over dabigatran; 2 subgroups favoring apixaban over rivaroxaban; 1 subgroup favoring dabigatran over rivaroxaban; and 1 subgroup favoring rivaroxaban over dabigatran in terms of risk reduction of the primary endpoint. No subgroup favored warfarin and most dabigatran vs warfarin users favored neither drug. The variables that most influenced favoring one subgroup over another included Age, history of ischemic stroke, thromboembolism, estimated glomerular filtration rate, Race, and myocardial infarction.

**Conclusions:**

Among patients with AF treated with a NOAC or warfarin, a causal ML method identified patient subgroups with differences in outcomes associated with OAC use. The findings suggest that the effects of OACs are heterogeneous across subgroups of AF patients, which could help personalize the choice of OAC. Future prospective studies are needed to better understand the clinical impact of the subgroups with respect to OAC selection.

Atrial fibrillation (AF) is the most common cardiac arrhythmia encountered in clinical practice and is associated with five-fold risk of stroke.^1, 2^ Oral anticoagulants (OACs) can effectively reduce the risk of stroke by up to 70% and are recommended for 80% of AF patients.^3–5^ Over the last decade, there have been major advances in the availability of new anticoagulants, providing patients and clinicians a significant treatment choice. While the availability of treatment options is beneficial for patients, it is not always clear if there is a preferred treatment option for a given patient. There is limited evidence on the heterogeneity in treatment effects associated with various OAC options.^6–8^

Conventional comparative effectiveness of OACs is typically based on population average treatment effect (ATE); that is, the difference in mean of an outcome of interest between the treatment groups.^9, 10^ However, not all patients in a given treatment group benefit equally, and focusing on the population level ATE may obscure risks and benefits that accrue to subgroups.^11^ Basic subgroup analyses are typically conducted to examine whether the treatment effect is superior/inferior in prespecified subgroups categorized by a few baseline characteristics (eg, age < 65 and ≥65 years, gender, or race/ethnicity). However, predefined subgroup-based analyses may ignore additional underlying heterogeneity. To personalize and improve treatments selection, data driven methods are required to empirically identify naturally occurring subgroups of patients who may truly benefit from the treatment.

Machine learning (ML) methods such as causal tree-based recursive par titioning algor ithms have been pro-posed to identify subgroups that respond differently towards a treatment in experimental and observational data.^12–15^ Using a large cohort of patients with AF, we propose a causal ML method to identify patient clusters (subgroups) with similar baseline characteristics that differ in magnitude and sign of their treatment effects of the OACs: apixaban, dabigatran, rivaroxaban, and warfarin with respect to each other.

## Methods

This study uses deidentified patient data and was exempted from review by the Mayo Clinic Institutional Review Board. The study conforms to the strengthening the reporting of observational studies in epidemiology (STROBE) statement.^16^

### Study source and population

We analyzed claims of 34,569 new users (age ≥18 years) of OACs at standard dose with nonvalvular AF between 10/01/2010 and 11/29/2017 included in the OptumLabs Data Warehouse (OLDW).^17^ Patients were included in the sample if they had at least 1 year of continuous insurance coverage before their first dispensed OAC (index date) and an estimated glomerular filtration rate (eGFR) ≥15 mL/min/1.73 m^2^ at baseline. The period before the first prescription dispensation was defined as the baseline time window (BTW), which was used to capture baseline patient characteristics. Patients were followed from the date of initiation of OAC until the occurrence of an endpoint, disenrollment in health insurance plan, switch/discontinuation of the initial drug, end of study, or death, whichever came first. Patients were also required to have serum creatinine measurements in the 12 months prior to the first OAC prescription dispensation. eGFR was calculated using the Chronic Kidney Disease Epidemiology Collaboration (CKD-EPI) equation.^18^ Patients who used dabigatran 110 mg twice daily and rivaroxaban 10 mg once daily, doses which are not approved in the US for AF, were also excluded. See Figure 1 in the Supplement for the patient selection flow diagram.

### Covariates

Baseline covariates (Table I) were ascertained in the BTW. Comorbidities were captured by ICD-9/10-CM diagnosis codes in any position on claims occurring within the BTW.^19^

CHA_2_DS_2_ -VASc and HASBLED scores were calculated for each patient.^20, 21^ Indicators (yes/no) were created for procedures and prescription fills in the BTW. These baseline characteristics were defined by the presence of a claim with eligible diagnosis codes, procedure codes, or prescription fills. The absence of such claims was interpreted as the absence of a condition. No missing values were observed in the demographics and eGFR variables. Patients were assigned to renal function groups according to the eGFR as 15 to 30, 30 to 45, 45 to 60, 60 to 90, >90 mL/min/1.73 m^2.^

### Treatment

Four OACs – 3 nonvitamin K antagonist oral anticoagulants (NOACs: apixaban, dabigatran, and rivaroxaban) and warfarin were investigated. Fill dates and days supplied per prescription were used to determine treatment episodes, defined as the period from the fill date to the date when there were no residual days of supply. A maximum gap of 30 days between treatment episodes was allowed. The discontinuation date was the end of the last treatment episode plus 30 days.

### Endpoints

Our primary outcome was a composite of ischemic stroke, intracranial hemorrhage, and all-cause mortality, as it captures the most specific events representing highest severity and irreversibility.^22^ Secondary analyses were also performed for major bleeding and all-cause mortality. Mortality was determined using a combination of the Social Security Death Master File and discharge status. The other outcomes were defined as a primary diagnosis during an emergency room visit or an inpatient stay.

The data for this study have been previously reported in,^23^ and the list of ICD 9/10-CM diagnosis codes validation of the diagnosis codes, definition of outcomes, and inclusion and exclusion criteria can be found in the online supplementary material.

### Matching

For each pairwise (head-to-head) treatment comparison group, we applied a ML matching technique based on the random forest (RF) algorithm.^24^ Specifically, we selected the most relevant variables for predicting the treatment using the Boruta algorithm,^25^ and then use the variables to build a RF model to estimate propensity scores. The RF model also outputs a proximity matrix, which defines the similarity between treatment and control cases. Two patients are similar if they fall in the same terminal node of the model and dissimilar otherwise. For each treated patient, all control patients with propensity score within a small range were selected. Among these control patients, the patient(s) with the smallest distance from the treated patient was then selected.

### Statistical analyses

Descriptive statistics are reported with means (SDs) for continuous variables and counts (percentages) for categorical variables. The population and subgroup ATE are reported with 95% CI. Data management was performed using SAS 9.4 and data analyses performed using R version 3.5.1. R packages used for the analysis include ranger,^26^ tmle,^27^ Boruta,^25^ and causalTree.^28^

### Causal machine learning

We develop a novel hybrid causal tree (CT)^12^ and targeted maximum likelihood estimation (TMLE)^29^ method to discover patient subgroups characterizing the head-to-head treatment effects of the OACs on the endpoints. Specifically, we apply the CT algorithm to recursively partition the data into subgroups and use TMLE within the tree nodes to estimate ATE and associated confidence intervals (see supplementary methods for more details). The ATE quantifies the additive effect of an OAC on the endpoint if all patients in the subgroup were treated with the OAC compared to when treated with another OAC.

### Training and validation

To avoid overfitting and improve interpretability, we stop growing the tree when the depth exceeds a maximum (*max_depth* = 6), the number of treated or control observations (*n*_*min*_) and the number of events (*e*_*min*_) in a terminal node drops below a threshold (*n*_*min*_ = 100, *e_min_* = 8).^30^

### Optimal number of subgroups

**Figure 1:**
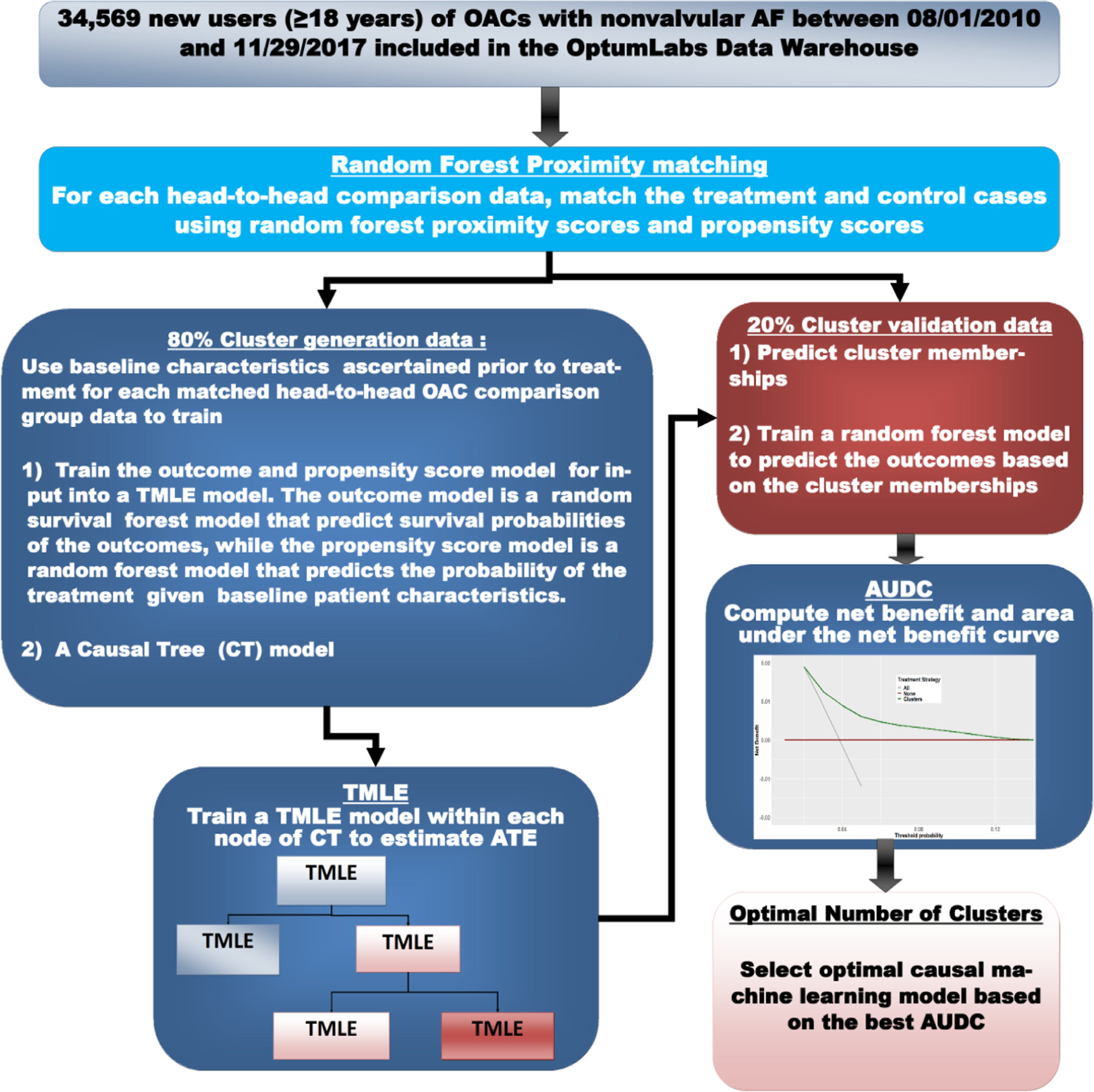
Training and validation of causal machine learning model. Workflow of the repeated training and validation procedure for hybrid causal machine model consisting of the CT, TMLE, and net benefit modules. Each matched head-to-head OAC comparison data are randomly divided into 2 parts: 80% used for developing the clustering and estimating ATE and 20% used to validate the clusters. *ATE*, average treatment effect; *CT*, causal tree; *OAC*, oral anticoagulants; *TMLE*, targeted maximum likelihood estimation.

We implemented a repeated training-validation approach, where the data was split into 2 parts: one part (80%) for building the causal ML model, and the second part (20%) for selecting the optimal number of subgroups. Specifically, using the causal ML model developed on the 80% portion, we predict subgroup memberships on the 20% portion and then train a RF model based on the subgroup memberships to predict an endpoint. We also compute the net benefit of the RF model.^31–33^ The net benefit reflects the clinical utility of using the subgroupings to make clinical decisions.^31–33^ The benefit-harm relationship of the different subgroupings can be illustrated by a decision curve,^31–33^ which is a plot of the net benefit across all possible risk thresholds. The area under the decision curve (AUDC) provides an overall accuracy measure that can be used to select the best causal ML.^34^ Figure 1 provides a workflow of the repeated training and validation procedure.

### Head-to-head ATE

The causal ML model generates head-to-head (OACs: *A vs B*) ATEs, which are expressed as the ATE of the first drug *A* over the second drug *B*. A negative value indicates that drug *A* is associated with a lower risk of an outcome compared to drug *B*. For example, in comparing the effectiveness of apixaban vs dabigatran, an ATE of −0.05 is interpreted as: “apixaban reduces the (absolute) risk of the outcome by 5% over dabigatran.”^29, 35^ The ATE value at the root node (Figures 4 – 10) represents the marginal treatment effect of drug *A* over drug *B* at the population level.

## Results

### Characteristics

The second column of Table I presents descriptive statistics prior to matching for all 34,569 AF patients. The mean age, number of females and whites was 71.2 (SD, 10.7) years, 14,916 (43.1%), and 25,051 (72.5%) respectively. The mean follow-up for all the endpoints was 8.3 (SD, 9.0) months. During follow-up, 2,110 (6.1%) of patients experienced the primary outcome, 1,675 (4.8%) died, and 1,068 (3.1%) had major bleeding. The number of patients who used apixaban, dabigatran, rivaroxaban, and warfarin was 11,350 (32.8%), 3,435 (9.9%), 8,597 (24.9%), and 11,187(32.4%), respectively. The last 4 columns of Table I present baseline characteristics categorized by OAC use prior to matching. Apixaban and warfarin users were older, while dabigatran users were younger. Warfarin users tended to have higher HASBLED scores, more comorbid conditions, moderate to severe renal insufficiency (eGFR = 5–60 mL/min/1.73 m^2^), and higher rates of the primary and secondary endpoints. Table 2 in the Supplement presents baseline descriptive statistics after matching for each of the head-to-head OAC treatment groups.

### Matched event rates per 1,000 person-years

**Figure 2:**
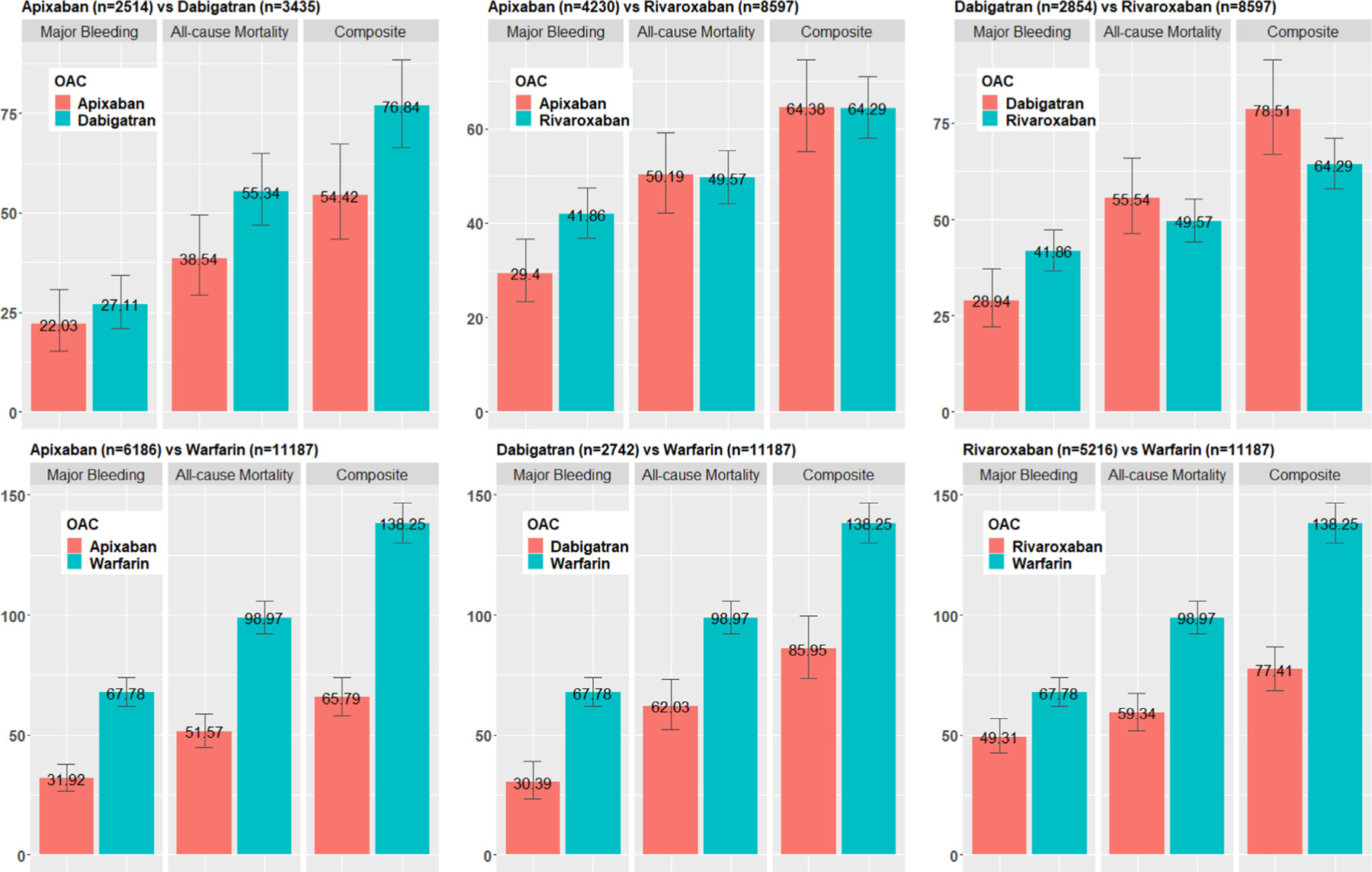
Matched event rates of major bleeding, All-cause mortality, and the composite endpoint. All numbers were calculated using matched head-to-head OAC comparison groups. Event rates were calculated as number of events per 1,000 person-year. *OAC*, oral anticoagulants.

Among 11,350 users of apixaban, 2,514 were matched with 3,435 dabigatran users. Event rates (per 1,000 per son-year s, Figure 2) for the primary outcome (54.42 vs 76.84), all-cause mortality (38.54 vs 55.34) and major bleeding (22.03 vs 27.11) were lower for apixaban users compared to dabigatran. About 4,230 apixaban users were matched to 8,597 rivaroxaban users, in which, the event rates for the primary outcome were similar (64.38 vs 64.29). Total 11,421 optimal matched pairs were obtained for dabigatran vs rivaroxaban. Event rates for the primary outcome (78.51 vs 64.29) and all-cause mortality (53.54 vs 49.57) were higher for dabigatran compared to rivaroxaban users, while dabigatran users had lower major bleeding rates (28.94 vs 41.86). About 11,187 warfarin users were matched to 6,186 apixaban, 2,742 dabigatran, and 5,216 rivaroxaban users. Event rates for warfarin users were higher compared to the other OACs.

### Population ATE

**Figure 3:**
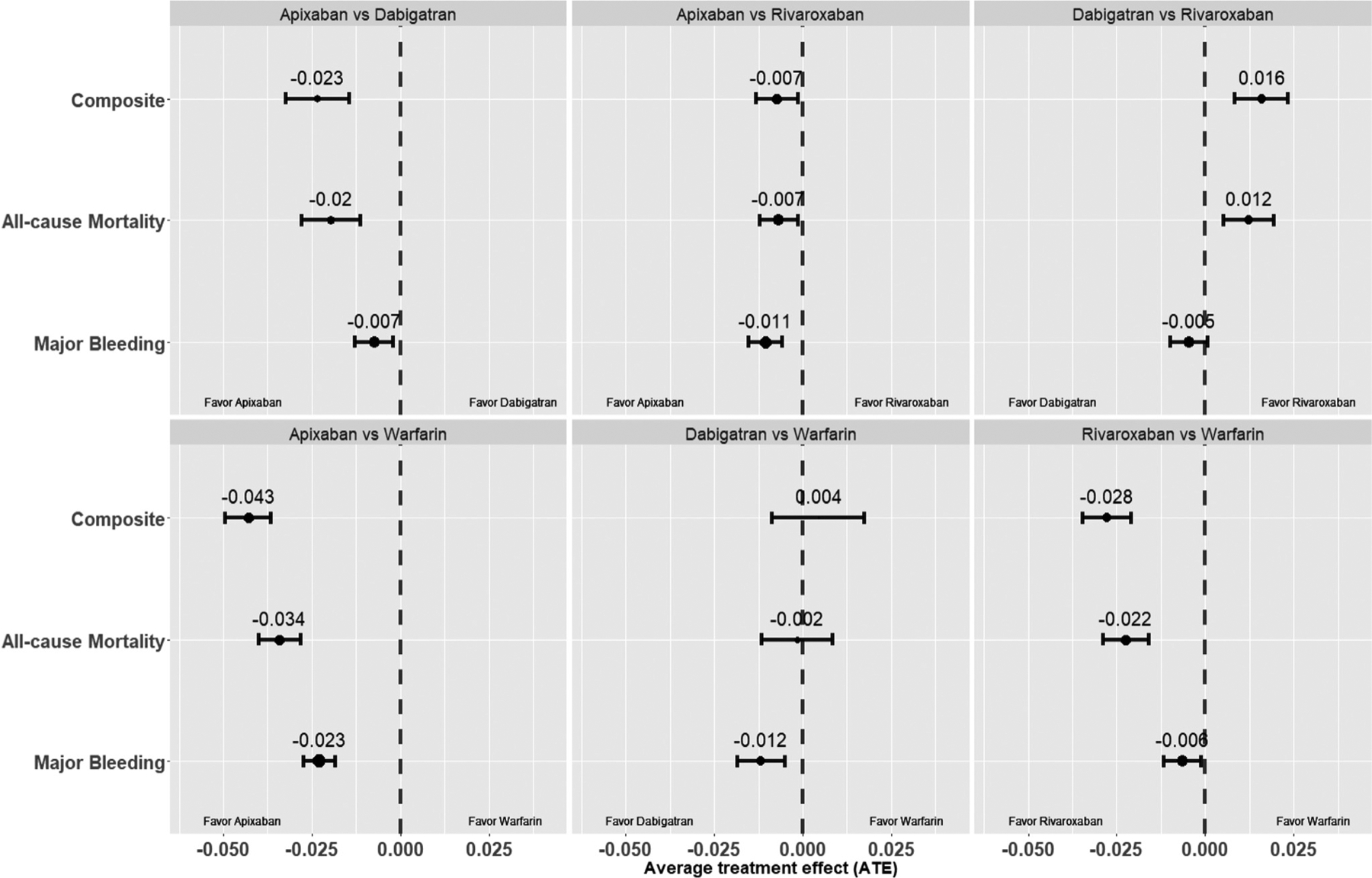
Population ATE. Population level average treatment effects of the OACs, apixaban, dabigatran, rivaroxaban, and warfarin on the endpoints, major bleeding, all-cause mortality, and the composite endpoint. All values were calculated using matched head-to-head OAC comparison groups. *OAC*, oral anticoagulants.

Figure 3 (and Table 3 in the Supplement) show the population level head-to-head ATE of the OACs on the primary and secondary outcomes. Apixaban (ATE, −0.04 [−0.05, −0.04]) and rivaroxaban (ATE, −0.03 [−0.04, −0.02]) were associated with lower risk of the primary outcome compared to warfarin, while dabigatran and warfarin were similar (ATE, 0.00 [−0.01, 0.02]).

Among NOACs, apixaban was associated with lower risk of the primary outcome compared to dabigatran (ATE, −0.02 [−0.03, −0.01]) and rivaroxaban (ATE, −0.01 [−0.01, −0.001]), while dabigatran was associated with higher risk (ATE, 0.02 [0.01, 0.02]) compared to rivaroxaban.

### Subgroup ATE

**Figure 4:**
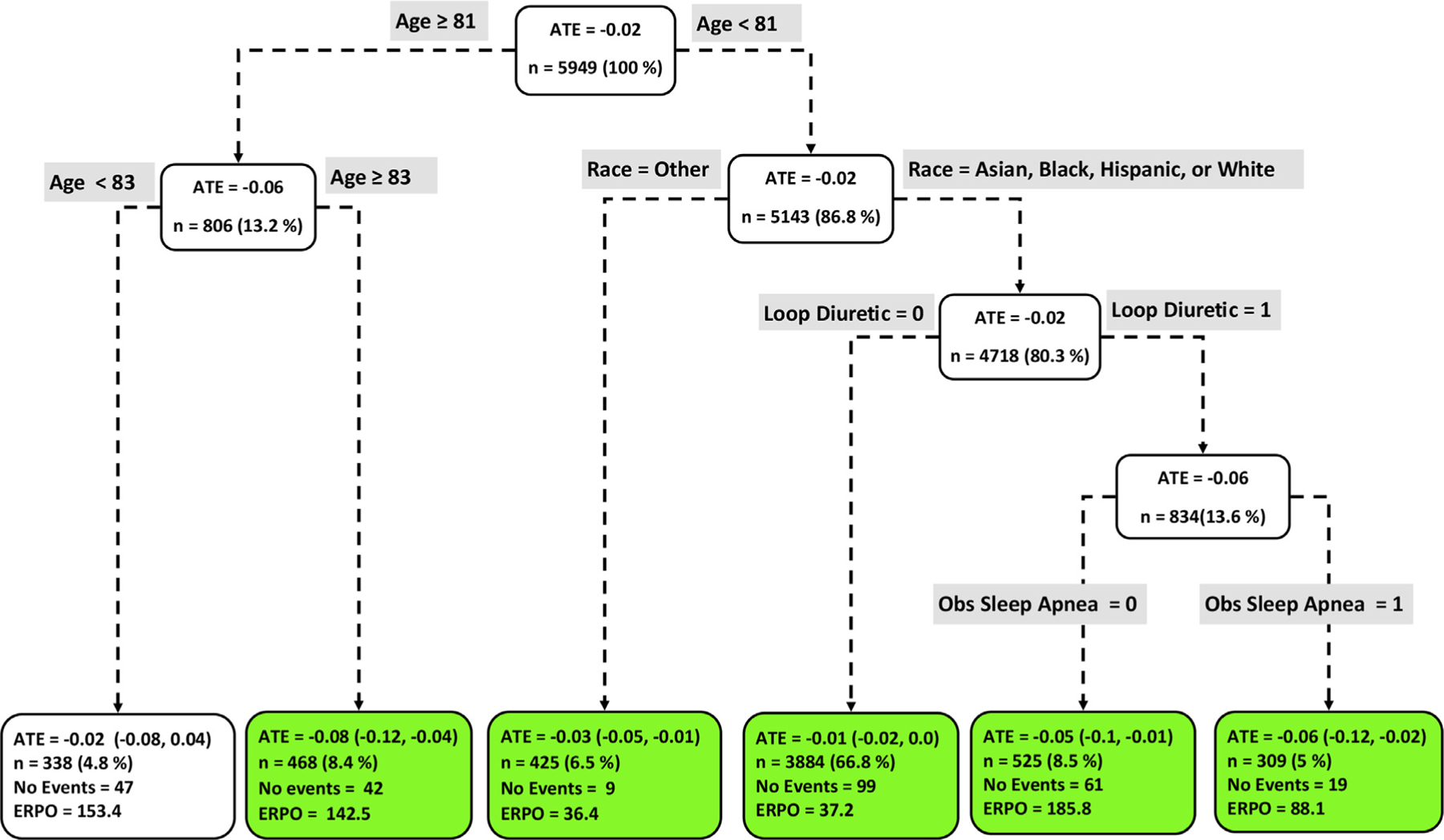
Subgroups of apixaban vs dabigatran users with respect to the primary composite endpoint. The subgroups are the terminal nodes of the optimal causal ML model. The green subgroups favor the use of apixaban. All the values were estimated based on the matched sample of apixaban and dabigatran users. *ATE* indicates average treatment effect; *ERPO*, events rate per 1,000.

**Figure 5:**
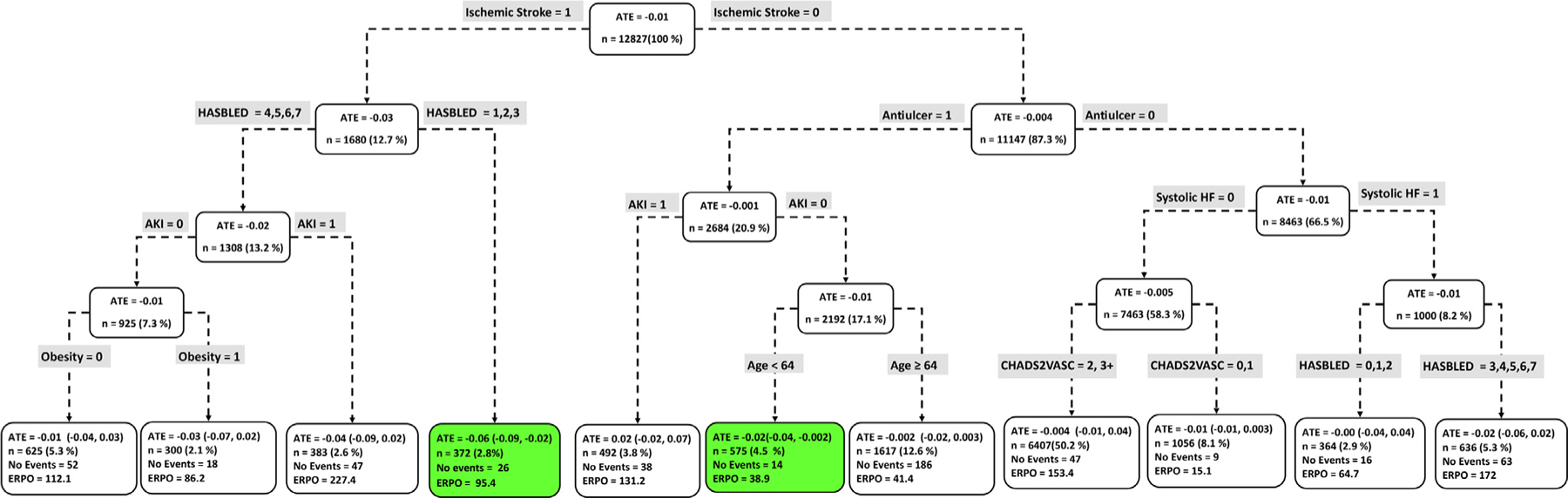
Subgroups of apixaban vs rivaroxaban users with respect to the primary composite endpoint. The subgroups are the terminal nodes of the optimal causal forest model. The green subgroups favor the use of apixaban. All the values were estimated based on the matched sample of apixaban and rivaroxaban users. *ATE*, average treatment effect; *ERPO*, events rate per 1,000.

**Figure 6:**
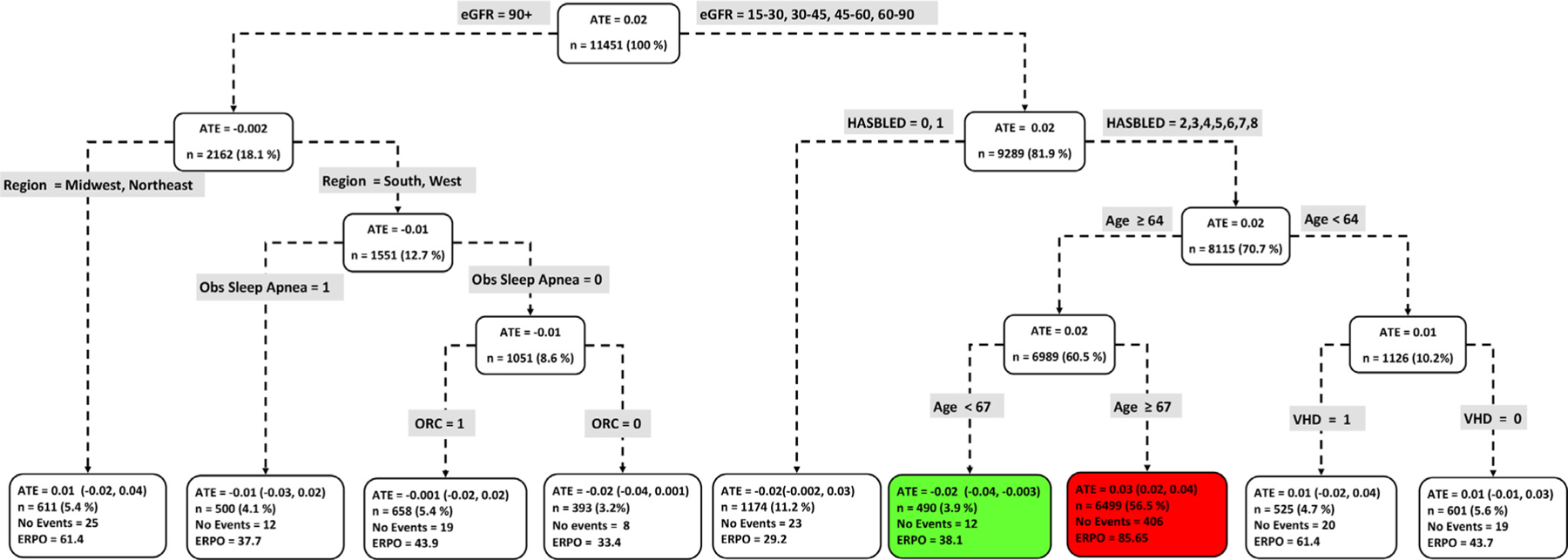
Subgroups of dabigatran vs rivaroxaban users with respect to the primary composite endpoint. The subgroups are the terminal nodes of the optimal causal forest model. The green subgroup favors the use of dabigatran while the red subgroup favors rivaroxaban. All the values were estimated based on the matched sample of dabigatran and rivaroxaban users. *ATE*, average treatment effect; *ERPO*, events rate per 1,000.

**Figure 7:**
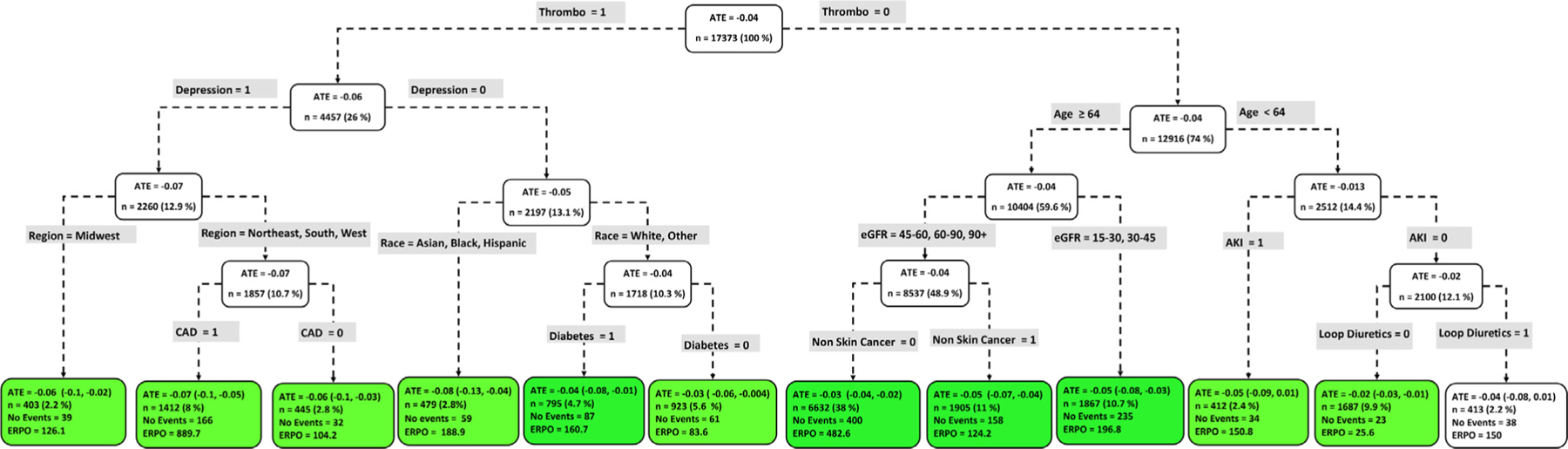
Subgroups of apixaban vs warfarin users with respect to the primary composite endpoint. The subgroups are the terminal nodes of the optimal causal forest model. The green subgroups favor the use of apixaban. All the values were estimated based on the matched sample of apixaban and warfarin users. *ATE*, average treatment effect; *ERPO*, events rate per 1,000.

**Figure 8:**
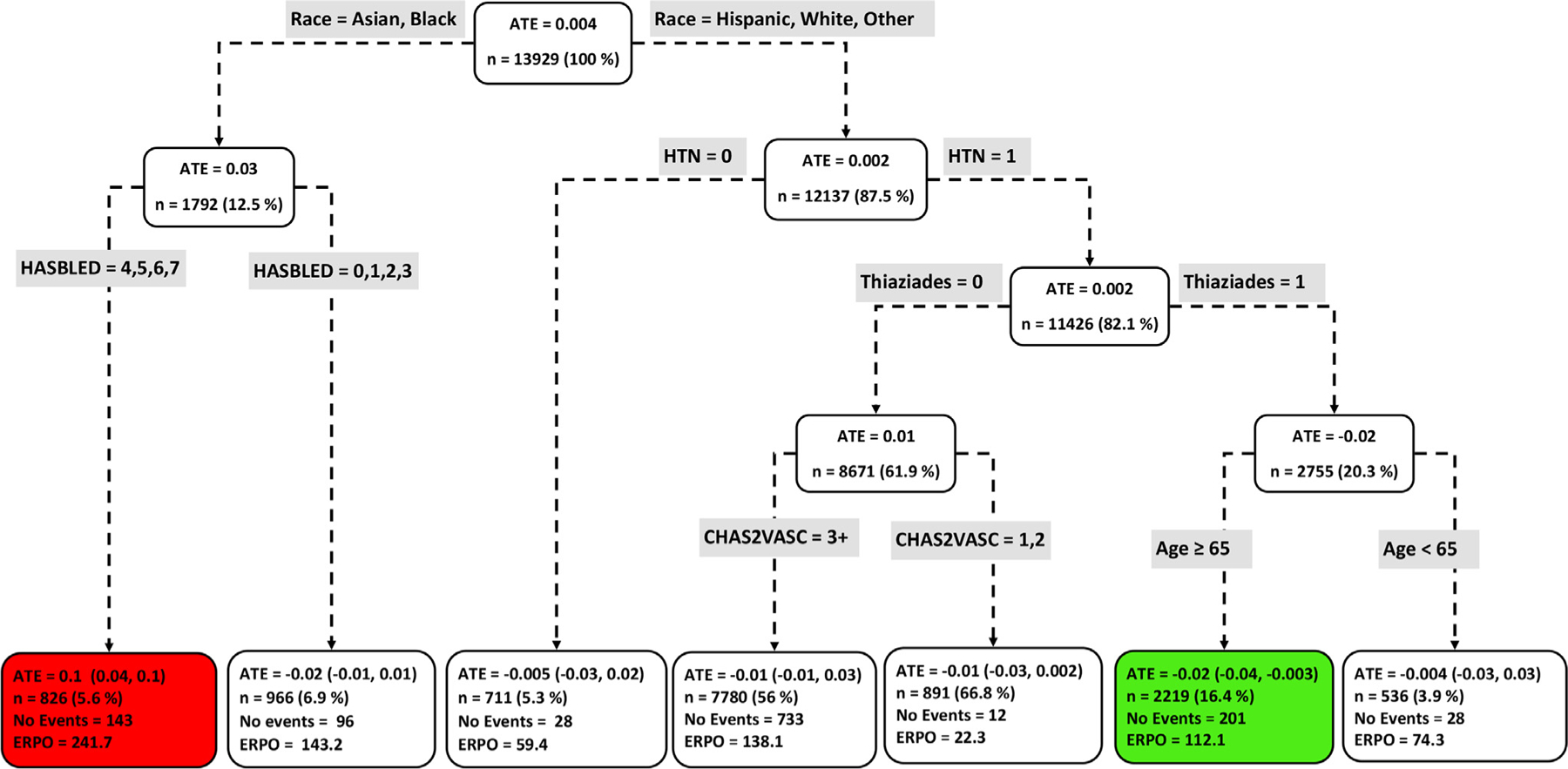
Subgroups of dabigatran vs warfarin users with respect to the primary composite endpoint. The subgroups are the terminal nodes of the optimal causal ML model. The green subgroup favors the use of dabigatran while the red subgroup favors warfarin. All the values were estimated based on the matched sample of dabigatran and warfarin users. *ATE*, average treatment effect; *ERPO*, events rate per 1,000; *ML*, machine learning.

**Figure 9:**
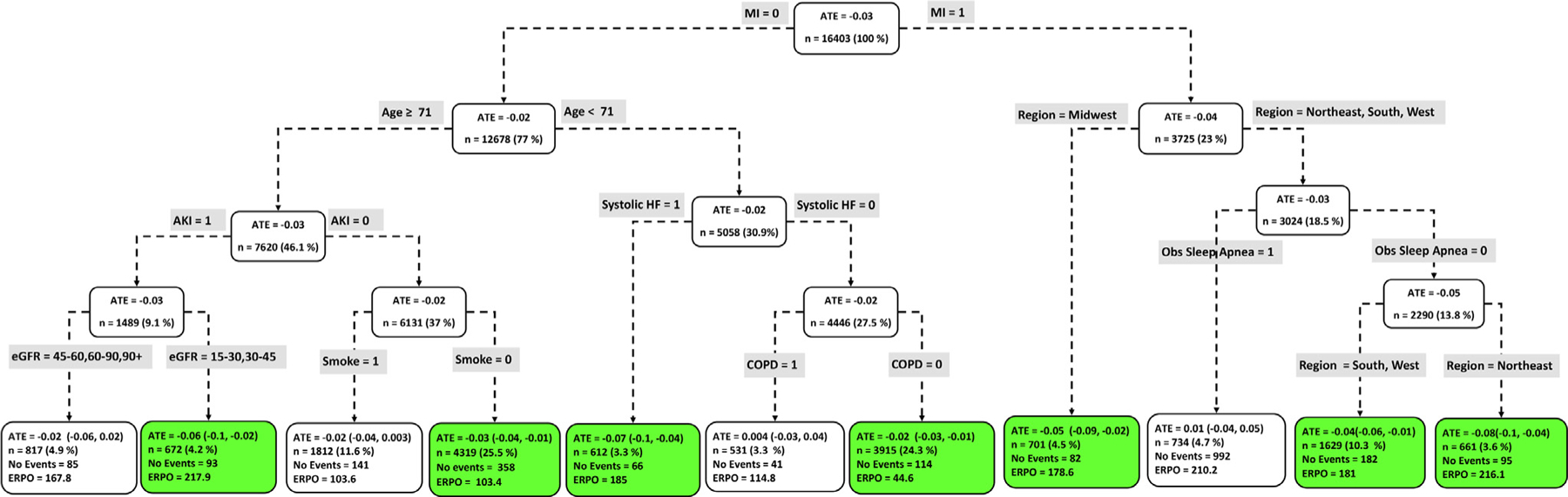
Subgroups of rivaroxaban vs warfarin users with respect to the primary composite endpoint. The subgroups are the terminal nodes of the optimal causal ML model. The green subgroups favor the use of rivaroxaban. All the values were estimated based on the matched sample of rivaroxaban and warfarin users. *ATE*, average treatment effect; *ERPO*, events rate per 1,000; *ML*, machine learning.

Figures 4 to 9 show the optimal causal ML tree structure for each head-to-head comparison groups, with clusters or subgroups represented by the terminal nodes. The ATE and its 95% confidence interval (CI), subgroup sizes (%), event rate, and event rate per 1,000 person-years (ERPO) are shown in the terminal nodes. The root node ATE represents the population ATE, which was depicted in Figure 3. Table 4 in the Supplement presents the subgroup results in Figures 4 to 6 in tabular form with maximum follow-up time.

In Figures 4 to 9, the variables considered for the split at the root node are most influential in favoring one subgroup over another, and included Age, history of ischemic stroke, thromboembolism, estimated glomerular filtration rate (eGFR), race, and myocardial infarction.

### Apixaban vs dabigatran

Figure 4 shows the causal ML tree structure for apixaban vs dabigatran. Five subgroups of apixaban vs dabigatran users demonstrated characteristics favoring apixaban. The largest subgroup (n = 3,884) of patients who benefitted from taking apixaban is described by age < 81 years, Asian, Black, Hispanic, or White, and not prescribed Loop Diuretic. Overall, patients who benefitted from taking apixaban can be described as age < 81 or age ≥ 83 years old. One subgroup (age between 81 and 83) with 338 patients showed no beneficial effect with respect to the use of either drug.

### Apixaban vs rivaroxaban

Figure 5 shows the causal ML tree structure for apixaban vs rivaroxaban. Defining patient characteristics in 2 subgroups, a subgroup with 372 patients described by ischemic stroke and HASBLED score ≤ 3 and a subgroup with 575 patients described by no ischemic stroke, antiulcer, no acute kidney injury (AKI) and age < 64 favored apixaban over rivaroxaban. No subgroup favored rivaroxaban.

### Dabigatran vs rivaroxaban

Nine subgroups of dabigatran vs rivaroxaban users showed differential beneficial effects of either drug with respect to the composite endpoint. From Figure 3, while rivaroxaban was favored at the population level, Figure 6 however shows that there exists a subgroup (n = 490) where dabigatran was favored over rivaroxaban (ATE, −0.02 [−0.043, −0.003]). Patient factors favoring dabigatran over rivaroxaban include less favorable renal function (eGFR = 5–30, 30–45, 45–60, 60–90), HASBLED score ≥2 and age between 64 and 67 years old. Interestingly, the same patient factors, except for older patients (age ≥ 67 years old) also favored the use of rivaroxaban.

### NOACs vs warfarin

Among NOAC vs warfarin users, we identified patient subgroups with differential benefits of the drugs compared with respect to the composite endpoint. Apixaban was uniformly favored across all subgroups (Figure 7) except for a subgroup of 413 patients, characterize by no history of thromboembolism, Age < 64, no AKI, and prescribed loop diuretic, where neither drug was favored. In contrast, Figure 8 shows that the majority (7 subgroups) of dabigatran vs warfarin users favored neither drug, with only one subgroup (n = 2,219) favoring dabigatran (ATE, −0.02 [−0.044, −0.003]) and 1 subgroup (n = 826) favoring warfar in (ATE, 0.10 [0.04, 0.16]). Patient factors favoring warfarin use include Black or Asian and high HASBLED score (≥3). Of 11 rivaroxaban vs warfarin subgroups, rivaroxaban was favored in 7 subgroups, while neither drug was favored in 4 subgroups (Figure 9). The smallest subgroup favoring rivaroxaban (n = 612) is described by patients with no history of myocardial infarction, age < 71, and a history of systolic heart failure.

### Secondary outcomes

Figures 2 to 13 in the Supplement present results for the head-to-head ATEs on major bleeding and all-cause mortality. Equally, key patient factors can be seen to be associated with favoring one OAC over another. Table 5 in the Supplement presents the subgroup results shown in these figures in tabular form with maximum follow-up time.

## Discussion

### Methodology

We demonstrated the application of a suite of data-driven ML techniques to uncover heterogeneous treatment effects of OACs in AF patients. Our methodological approach consisted of: (1) apply a novel ML matching approach based on propensity score and proximity matrix estimated by the RF algorithm to match treatment and control observations, (2) apply the causal tree (CT) algorithm to partition study data into subgroups driven by differences in treatment assignments, (3) apply the well-established double robust TMLE method to estimate ATE within the nodes of CT, and finally (4) apply net benefit analysis to select the optimal number of subgroups based on how well the subgroups predict the endpoints. By first matching the data before application of a data clustering technique, we can help prevent false discovery of heterogeneous treatment effects.^36^ We combined all these analytic techniques, which capitalizes on efficient use of the random forest methodology to discover novel patient subgroups with heterogeneous responses to OACs.

Unlike other ML methods, which may generate excellent performance results but are difficult to interpret, human experts can interpret the tree structure of our causal model: each identified subgroup is defined by a handful of baseline characteristics, which could facilitate treatment decisions.

### Population ATE

Among AF patients, risk of the outcomes was lower among apixaban, and rivaroxaban users compared to warfarin users. Dabigatran was associated similar risk of the primary outcome and all-cause mortality, but a lower risk of major bleeding compared to warfarin. The population ATEs for the secondary outcomes are largely parallel results from previously published observational studies,^9, 23, 37–39^ including a recent study by our group using the same data set based on propensity score adjustment and the cox proportional hazard regression model. Among the NOACs, apixaban was favored compared to dabigatran and rivaroxaban in reducing risk of all the outcomes. Dabigatran was favored in reducing risk of major bleeding compared to rivaroxaban, while rivaroxaban was favored in reducing risk of the primary and all-cause mortality. Our results for all-cause mortality were different from our previous findings,^23^ which found no difference in risk among the NOACs. However, these studies have several differences with respect to population adjustments and analytic methods used. These results emphasize the need to consider different data driven approaches in investigating the effects of interventions.

### Heterogeneous treatment effects

In contrast to the population level results, where apixaban or rivaroxaban were associated with lower risk (Figure 3) compared to warfarin, we discovered several subgroups for which the use of either drug (apixaban or warfarin, rivaroxaban, or warfarin) was associated with no risk reduction (Figures 7 and 9). It is possible that these patients might benefit from some other treatment option, which can be predicted by our approach. Similarly, while dabigatran and warfarin were associated with similar risks at the population level, we discovered subgroups of patients with unique characteristics favoring either dabigatran or warfarin. Amongst NOAC users, we identified important patient factors favoring one NOAC over another. Overall, more apixaban subgroups were favored compared to dabigatran and rivaroxaban subgroups. These findings indicate that outcome differences between NOACs and warfarin users are heterogeneous across different AF subgroups, and we were able to identify defining patient characteristics through application of a novel data driven and interpretable causal ML technique.

### Clinical implications in AF

Current practice guidelines recommend NOACs over warfarin for the demonstrated risk reduction in intracranial bleeding.^9, 40–44^ In our study, we found only a handful of subgroups that may benefit from warfarin, which may further support the current guideline recommendations. The guidelines do not specifically recommend one NOAC versus another, but in all comparisons involving apixaban, we found that either apixaban was the preferred medication or none of the compared medications was preferred over the other. Apixaban and rivaroxaban have become the most commonly prescribed medications, largely due to apixaban’s lower risk of bleeding and rivaroxaban’s convenient once daily regimen.^9, 10, 37^ However, the estimated benefit-harm profiles are based on the entire population, which apply essentially to the “average” patient. Given, there is no average patient; patients often show significant heterogeneity in responses to treatments, and these average effects may not translate to individual patients. Understanding treatment heterogeneity is important for selecting the NOAC that will reduce risk of adverse outcomes for each individual patient. Most existing studies have focused on prespecifying subgroups categorized by one or two patient factors (eg, eGFR = 15–30, 30–45, 45–60, 60–90, >90 mL/min/1.73 m^2^ subgroups^23^) to address treatment effect heterogeneity. However, these predefined subgroup-based analyses ignore additional underlying heterogeneity, which we uncovered in this study. Our results suggest that OAC selection based on the data-driven subgroups we have identified could help improve outcomes for AF patients. However, the uncovered heterogeneity in this study have not been tested, additional prospective studies are required to understand the extent of the clinical benefit of the OAC subgroups.

### Study limitations

Our study has limitations. First, as with most studies based on observational data, our findings may be subject to residual confounding, selection bias, misclassification of the exposures and outcomes, and generalizability of study results. We attempted to address these issues with the use of validated algorithms for outcome and treatment definitions^36^ and appropriate use of data science methods. To overcome confounding, we followed a robust ML based matching approach to balance the treatment and control groups. The random forest model has been demonstrated to be capable of providing accurate and less model-dependent estimates of the propensity score as well as the proximity matrix we used for matching. However, even with this advanced data balancing technique, there is still the possibility of not balancing some pretreatment covariates (Table 6 in the Supplement). The application of double robust estimators such as the TMLE method can help guard against any residual cofounding and generate consistent estimate of the treatment effects.^45^ Despite our use of all of these state-of-the-art data science techniques, we acknowledge the limitations of ML methods in addressing causal relationships in large observational studies.^46, 47^

Second, clustering results from most ML algorithms critically depend on the underlying patient data, parameters of the algorithm, variables used for training, and the method to select the best number of clusters. Thus, the findings might differ if a different algorithm was used, or additional variables are included.

Finally, our study only examined the impact of OAC initiation on subgroups; we did not examine medication dosing. However, there are limited dosage forms available, and most patients receive the same dosages, unless they have renal insufficiency.

## Conclusion

We developed and applied a novel hybrid causal machine learning tool that uncovered heterogeneity in treatment effect of OACs by identifying subgroups of patients with AF who are more likely to benefit with one treatment as compared with another, or with no OAC. This approach could help personalize OAC selection among patients with AF; however, future prospective studies appear to be needed to better understand the extent of the OAC benefit across the different patient subgroups.

## Supplementary Material

Supplementary Material

## Figures and Tables

**Table I.** Baseline patient characteristics and outcomes for all patients and by OAC (prior to matching)

Variables	All patients	Apixaban	Dabigatran	Rivaroxaban	Warfarin
Sample size	34,569	1,350	3,435	8,597	11,187
Age (y) mean (SD)	71.2 (10.7)	72.3 (10.5)	67.2 (11.1)	69.4 (11.0)	72.8 (9.8)
CHADS2VASC, mean (SD)*					
0	553 (1.6)	139 (1.2)	106 (3.1)	217 (2.5)	91 (0.8)
1	1,880 (5.4)	548 (4.8)	330 (9.6)	649 (7.5)	353 (3.2)
2	3,604 (10.4)	1,131 (10.0)	532 (15.5)	1,174 (13.7)	767 (6.9)
3+	28,532 (82.5)	9,532 (84.0)	2,467 (71.8)	6,557 (76.3)	9,976 (89.2)
HASBLED, mean (SD)^†^					
0	679 (2.0)	196 (1.7)	128 (3.7)	232 (2.7)	123 (1.1)
1	3,400 (9.8)	999 (8.8)	558 (16.2)	1,112 (12.9)	731 (6.5)
2	8,348 (24.1)	2,685 (23.7)	954 (27.8)	2,355 (27.4)	2,354 (21.0)
3	10,342 (29.9)	3,382 (29.8)	974 (28.4)	2,527 (29.4)	3,459 (30.9)
4	7,510 (21.7)	2,576 (22.7)	547 (15.9)	1,571 (18.3)	2,816 (25.2)
5	3,320 (9.6)	1,147 (10.1)	230 (6.7)	637 (7.4)	1,306 (11.7)
6	845 (2.4)	327 (2.9)	35 (1.0)	139 (1.6)	344 (3.1)
7	119 (0.3)	36 (0.3)	NA	23 (0.3)	53 (0.5)
US region, n (%)					
Midwest	5,825 (16.9)	1,922 (16.9)	439 (12.8)	1,288 (15.0)	2,176 (19.5)
Northeast	6,046 (17.5)	1,912 (16.8)	558 (16.2)	1,586 (18.4)	1,990 (17.8)
South	19,306 (55.8)	6,498 (57.3)	2,091 (60.9)	4,840 (56.3)	5,877 (52.5)
West	3,392 (9.8)	1,018 (9.0)	347 (10.1)	883 (10.3)	1,144 (10.2)
Race, n (%)					
Asian	867 (2.5)	261 (2.3)	96 (2.8)	272 (3.2)	238 (2.1)
Black	3,483 (10.1)	1,246 (11.0)	272 (7.9)	719 (8.4)	1,246 (11.1)
Hispanic	2,259 (6.5)	714 (6.3)	212 (6.2)	621 (7.2)	712 (6.4)
White	25,051 (72.5)	7,987 (70.4)	2,614 (76.1)	6,287 (73.1)	8,163 (73.0)
Other	2,909 (8.4)	1,142 (10.1)	241 (7.0)	698 (8.1)	828 (7.4)
Gender: female, n (%)	14,916 (43.1)	5,376 (47.4)	1,251 (36.4)	3,427 (39.9)	4,862 (43.5)
Medical conditions, n (%)					
Heart failure (HF)	13,076 (37.8)	4,227 (37.2)	1,010 (29.4)	2,637 (30.7)	5,202 (46.5)
Hypertension (HTN)	32,141 (93.0)	10,560 (93.0)	3,104 (90.4)	7,865 (91.5)	10,612 (94.9)
Thromboembolism (Thrombo)	7,707 (22.3)	2,562 (22.6)	623 (18.1)	1,535 (17.9)	2,987 (26.7)
Diabetes mellitus	14,817 (42.9)	4,747 (41.8)	1,346 (39.2)	3,435 (40.0)	5,289 (47.3)
Coronar y artery disease (CAD)	19,548 (56.5)	6,312 (55.6)	1,822 (53.0)	4,393 (51.1)	7,021 (62.8)
Peripheral artery disease (PAD)	5,240 (15.2)	1,640 (14.4)	396 (11.5)	1,079 (12.6)	2,125 (19.0)
History of major bleeding	8,544 (24.7)	2,773 (24.4)	804 (23.4)	1,920 (22.3)	3,047 (27.2)
History of intracranial bleeding	729 (2.1)	256 (2.3)	66 (1.9)	131 (1.5)	276 (2.5)
Liver disease	5,424 (15.7)	1,874 (16.5)	522 (15.2)	1,328 (15.4)	1,700 (15.2)
Alcoholism	1,946 (5.6)	623 (5.5)	184 (5.4)	517 (6.0)	622 (5.6)
Obesity	12,013 (34.8)	4,214 (37.1)	1,125 (32.8)	3,082 (35.8)	3,592 (32.1)
Smoke	12,109 (35.0)	4,208 (37.1)	1,058 (30.8)	2,952 (34.3)	3,891 (34.8)
Falls	5,257 (15.2)	1,955 (17.2)	362 (10.5)	1,117 (13.0)	1,823 (16.3)
Acute kidney injury (AKI)	6,391 (18.5)	2,186 (19.3)	365 (10.6)	1,098 (12.8)	2,742 (24.5)
Other valvular heart disease (VHD)	17,378 (50.3)	5,700 (50.2)	1,711 (49.8)	4,081 (47.5)	5,886 (52.6)
Nonskin cancer	7,083 (20.5)	2,344 (20.7)	618 (18.0)	1,578 (18.4)	2,543 (22.7)
Recent major bleeding	219 (0.6)	60 (0.5)	21 (0.6)	29 (0.3)	109 (1.0)
Recent thromboembolism	1,912 (5.5)	598 (5.3)	134 (3.9)	329 (3.8)	851 (7.6)
Hyperlipidemia	30,961 (89.6)	10,113 (89.1)	3,074 (89.5)	7,624 (88.7)	10,150 (90.7)
Ischemic stroke	5,416 (15.7)	1,837 (16.2)	412 (12.0)	1,019 (11.9)	2,148 (19.2)
Myocardial infarction (MI)	6,703 (19.4)	2,115 (18.6)	519 (15.1)	1,371 (15.9)	2,698 (24.1)
COPD	5,741 (16.6)	1,755 (15.5)	456 (13.3)	1,295 (15.1)	2,235 (20.0)
Obstructive sleep apnea	7,904 (22.9)	2,690 (23.7)	853 (24.8)	2,051 (23.9)	2,310 (20.6)
Systolic heart failure (HF)	5,319 (15.4)	1,850 (16.3)	349 (10.2)	999 (11.6)	2,121 (19.0)
Cardioversion	4,070 (11.8)	1,530 (13.5)	505 (14.7)	1,127 (13.1)	908 (8.1)
Ablation	987 (2.9)	313 (2.8)	162 (4.7)	288 (3.4)	224 (2.0)
Pacemaker/ICD	4,558 (13.2)	1,581 (13.9)	384 (11.2)	991 (11.5)	1,602 (14.3)
PCI/CABG	6,793 (19.7)	2,081 (18.3)	571 (16.6)	1,365 (15.9)	2,776 (24.8)
Depression	13,273 (38.4)	4,550 (40.1)	1,203 (35.0)	3,144 (36.6)	4,376 (39.1)
Dementia	2,497 (7.2)	939 (8.3)	154 (4.5)	493 (5.7)	911 (8.1)
Hypothyroidism	11,469 (33.2)	3,863 (34.0)	1,095 (31.9)	2,690 (31.3)	3,821 (34.2)
Thyrotoxicosis	1,853 (5.4)	586 (5.2)	212 (6.2)	450 (5.2)	605 (5.4)
Ulcer in upper GI tract	2,159 (6.2)	697 (6.1)	204 (5.9)	475 (5.5)	783 (7.0)
Medications, n (%)					
Antiplatelet	4,237 (12.3)	1,386 (12.2)	364 (10.6)	916 (10.7)	1,571 (14.0)
NSAIDS	4,077 (11.8)	1,376 (12.1)	444 (12.9)	1,047 (12.2)	1,210 (10.8)
Amiodarone	4,022 (11.6)	1,367 (12.0)	364 (10.6)	857 (10.0)	1,434 (12.8)
Dronedarone	928 (2.7)	296 (2.6)	169 (4.9)	268 (3.1)	195 (1.7)
Other antiarrhythmic drugs (OAAD)	3,564 (10.3)	1,264 (11.1)	484 (14.1)	1,041 (12.1)	775 (6.9)
Digoxin	3,200 (9.3)	821 (7.2)	371 (10.8)	669 (7.8)	1,339 (12.0)
Diltiazem	6,153 (17.8)	2,064 (18.2)	626 (18.2)	1,533 (17.8)	1,930 (17.3)
Verapamil	613 (1.8)	161 (1.4)	68 (2.0)	152 (1.8)	232 (2.1)
Other rate control drugs (ORC)	22,733 (65.8)	7,630 (67.2)	2,171 (63.2)	5,515 (64.2)	7,417 (66.3)
Renin-angiotensin system					
Renin-angiotensin system antagonists (ACE/ARB)	18,577 (53.7)	6,201 (54.6)	1,816 (52.9)	4,488 (52.2)	6,072 (54.3)
Other calcium channel blockers (CCB)	7,934 (23.0)	2,704 (23.8)	721 (21.0)	1,864 (21.7)	2,645 (23.6)
Other adrenergic blocking (Obeta)	2,012 (5.8)	608 (5.4)	184 (5.4)	437 (5.1)	783 (7.0)
Loop diuretics	8,582 (24.8)	2,831 (24.9)	649 (18.9)	1,718 (20.0)	3,384 (30.2)
Thiazides	8,053 (23.3)	2,692 (23.7)	858 (25.0)	1,956 (22.8)	2,547 (22.8)
Cholesterol-lowering drugs (Statins)	17,950 (51.9)	6,089 (53.6)	1,704 (49.6)	4,257 (49.5)	5,900 (52.7)
Insulin	2,842 (8.2)	894 (7.9)	233 (6.8)	574 (6.7)	1,141 (10.2)
Metformin	5,289 (15.3)	1,731 (15.3)	523 (15.2)	1,366 (15.9)	1,669 (14.9)
Other diabetes mellitus drugs (Odiab)	4,538 (13.1)	1,468 (12.9)	426 (12.4)	1,050 (12.2)	1,594 (14.2)
Antiulcer agents (Antiulcer)	9,233 (26.7)	3,189 (28.1)	774 (22.5)	2,034 (23.7)	3,236 (28.9)
eGFR groups, n (%)					
15–30	1,060 (3.1)	327 (2.9)	39 (1.1)	111 (1.3)	583 (5.2)
30–45	3,433 (9.9)	1,213 (10.7)	222 (6.5)	607 (7.1)	1,391 (12.4)
45–60	6,716 (19.4)	2,278 (20.1)	594 (17.3)	1,523 (17.7)	2,321 (20.7)
60–90	18,037 (52.2)	5,916 (52.1)	1,877 (54.6)	4,764 (55.4)	5,480 (49.0)
90+	5,323 (15.4)	1,616 (14.2)	703 (20.5)	1,592 (18.5)	1,412 (12.6)
Outcomes, n (%)					
Major bleeding	1,068 (3.1)	239 (2.1)	68 (2.0)	246 (2.9)	515 (4.6)
All-cause mortality	1,675 (4.8)	365 (3.2)	152 (4.4)	306 (3.6)	852 (7.6)
Primary composite	2,110 (6.1)	471 (4.1)	193 (5.6)	381 (4.4)	1,065 (9.5)
Follow-up time (mo), mean (SD)					
Follow up major bleeding	8.1 (8.9)	7.7 (7.7)	8.8 (10.8)	8.2 (9.0)	8.2 (9.4)
Follow-up all-cause mortality	8.7 (9.7)	7.9 (7.8)	9.6 (11.9)	8.6 (9.4)	9.2 (10.7)
Follow-up primary composite	8.1 (9.0)	7.7 (7.7)	8.8 (10.8)	8.3 (9.0)	8.3 (9.5)
Mean follow-up, all outcomes	8.3 (9.0)	7.7 (7.7)	9.0 (10.9)	8.4 (9.0)	8.6 (9.5)

CABG, coronary artery bypass graft; COPD, chronic obstructive pulmonary disease; eGFR, glomerular filtration rate; GI, gastrointestinal; ICD, implantable cardioverter-defibrillator; NA, counts that are less than 10, and we masked those cells in accordance with OptumLabs data use policy; NSAID, nonsteroidal anti-inflammatory drug; PCI, percutaneous coronary intervention.

Unless otherwise noted, data are presented as n (%) for categorical variables and mean (SD) for continuous variables.

* The CHADS2VASC score ranges from 0 to 9; higher score indicates higher risk of stroke. A point score is calculated as 1 point each for heart failure, hypertension, diabetes mellitus, vascular disease, age 65 to 74 years, and female sex; 2 points for age ≥75 y and prior stroke, TIA, or thromboembolism.

† The HASBLED score ranges from 0 to 9; higher score indicates higher risk of bleeding. A point score is calculated as 1 point each for hypertension, abnormal kidney function, abnormal liver function, prior stroke, prior bleeding or bleeding predisposition, labile international normalized ratio (INR), older than 65 years, medication usage predisposing to bleeding, and alcohol use. This study did not consider INR, so the range of HASBLED was 0 to 8.
