# Supplementary material for: Identifying treatment heterogeneity in atrial fibrillation using a novel causal machine learning method
Source: Am Heart J. Author manuscript; Available in PMC 2024 Jun 1. (PMC10615250; doi:10.1016/j.ahj.2023.02.015)
Supplement: Supplementary Material [file NIHMS1938588-supplement-Supplementary_Material.docx]

**Supplemental Material**

**Contents**

1. **Supplemental Methods:**
   1. Study population, inclusion and exclusion criteria
   2. Primary composite outcome
   3. Validation of diagnosis codes and definition of major bleeding
   4. Random Forest proximity matching
   5. A hybrid causal machine learning algorithm
   6. Training and validation
   7. Diagnosis codes for outcomes
2. **Supplementary Results**
   - **Table 2:** Baseline characteristics by head-to-head OAC treatment groups after matching
   - **Table 3:** Population ATE.
   - **Figures 2 – 7:** Tree structure for the head-to-head OAC comparison groups showing treatment effects on major bleeding
   - **Figures 8 – 13**: Tree structure for the head-to-head OAC comparison groups showing treatment effects on all-cause mortality
   - **Table 4:** Subgroup treatment effects of OACs on the primary composite outcome
   - **Table 5:** Subgroup treatment effects of OACs on the secondary outcomes.
   - **Table 6:** Apixaban vs. Dabigatran: Baseline characteristics pre-and post-matching

**Supplemental Methods**

1. Study population, inclusion and exclusion criteria

This study was a retrospective cohort analysis using the OptumLabs Data Warehouse, which contains data on patients with private insurance or Medicare Advantage of all ages and races throughout the US.^1^ The study population included adult patients (≥18 years) with nonvalvular AF who were newly prescribed one of four of oral anticoagulants (OACs), that is, warfarin and three NOACs (apixaban, dabigatran, and rivaroxaban), between October 1, 2010 and November 29, 2017 and had an estimated glomerular filtration rate (eGFR) ≥15 mL/(min·1.73 m2) at baseline. We did not consider the oral anticoagulant, edoxaban because of the very small number of users in the data. Patients were required to have linked serum creatinine data in the 12 months before the first OAC prescription dispensation, and the most recent creatinine value was used as baseline value. The patient selection flow diagram is shown in **Figure 1** (more details can be found in our prior work ^2^)


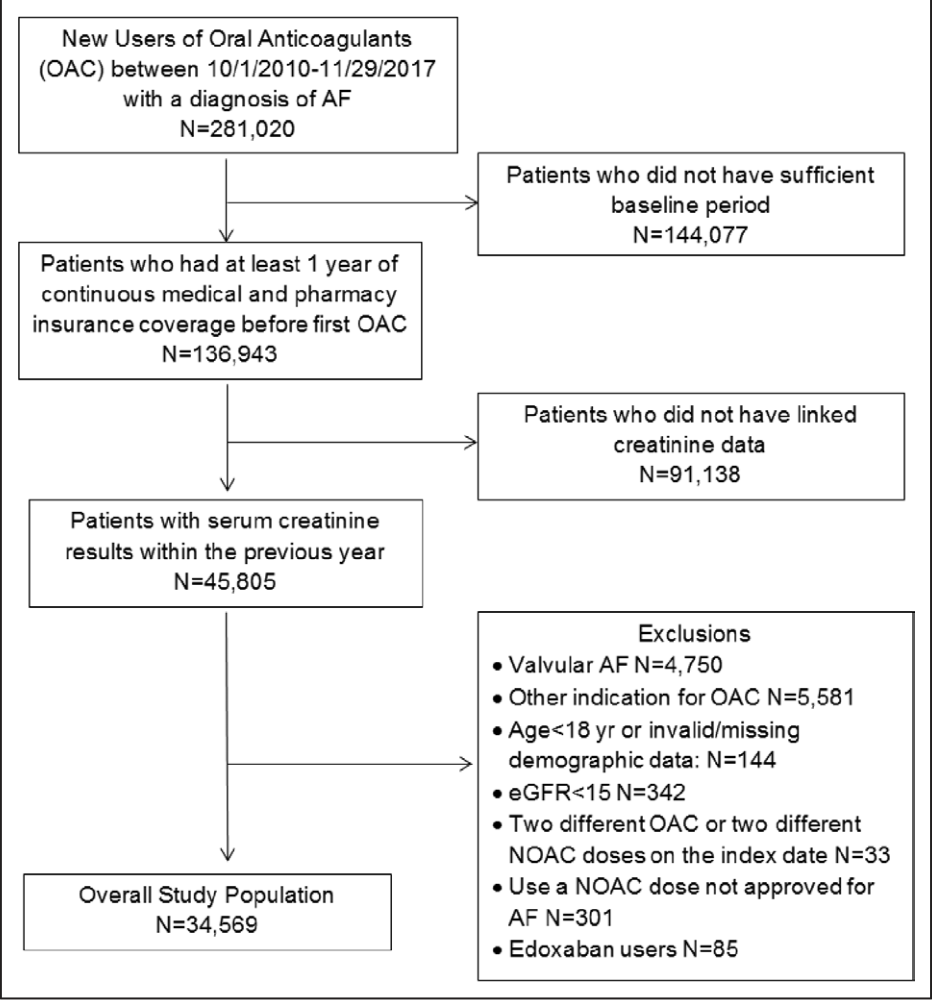


Figure 1: Patient selection flow diagram

1. Primary composite outcome

We take as primary outcome, ischemic stroke or systemic embolism, intracranial bleeding and all-cause mortality. That is, the primary endpoint for the analysis was the occurrence of any of ischemic stroke or systemic embolism, intracranial bleeding and all-cause mortality. This outcome was chosen as it captures the most specific events representing highest severity and irreversibility.^3^

1. Validation of Diagnosis Codes and Definition of Major Bleeding

The diagnosis codes and algorithms used to ascertained outcomes and comorbidities in this study have been commonly used and validated in many previous studies.^4-7^ We also leveraged our ability to link claims data to laboratory results and electronic health records to validate diagnosis codes.

We conducted internal validation of major bleeding based on the International Society on Thrombosis and Haemostasis (ISTH) criteria^8^: (1) Fatal bleeding, and/or, (2) Symptomatic bleeding in a critical area or organ, such as intracranial, intraspinal, intraocular, retroperitoneal, intraarticular or pericardial, or intramuscular with compartment syndrome, and/or, (3) Bleeding causing a fall in hemoglobin level of 2 g/dL or more, or leading to transfusion of two or more units of whole blood or red cells. We used ICD-9 and CPT procedure codes to identify transfusion, but we were not able to know the units of whole blood or red cells used in the transfusion. We also identified other procedures to control bleeding, such as endoscopic procedures to address gastrointestinal bleeding, neurosurgical decompression for intracranial bleeding, evacuation of hematoma, or vascular embolization procedures to control bleeding. Among all bleeding events, one in four was bleeding in critical areas, and one third required transfusion. This is generally consistent with previous studies that adapted ISTH definition using administrative data.^9^

Additional details on the diagnosis codes, definition of outcomes, inclusion and exclusion criteria, the overall study population and data source can be found in our prior study,^2^ as well as the online supplementary material (<https://www.ahajournals.org/doi/suppl/10.1161/CIRCOUTCOMES.120.006515> ).

1. Matching with propensity score and Random Forest proximity scores

*Random Forest*

The random forest algorithm (RF) is an ensemble learning method where multiple regression and classification trees are generated on a random sample of observations and predictors from the data and the predictions combined by averaging (for regression problems) or majority vote (for classification problems).^10^ The randomness introduced at the observation and variable levels in RF introduces a form of regularization that can result in a flexible and robust model. Further, by combining the predictions from the randomly generated decision trees, RF encourages diversity in the model, which has been recognized as a key to generating accurate and stable models.

*Variable selection for propensity score model using Boruta algorithm*

The goal of propensity score (PS) analysis is to make baseline patient characteristics comparable between the treatment groups as in randomize control trial. Thus, it is generally recommended to include all potential confounders in the PS model. However, in large databases (many variables), it is often difficult to decide which variable is a potential confounder or not. However, we know that a true confounding variable is strongly associated with the treatment (but not a proxy for the treatment), and the distribution of the confounding variable within the treatment groups is often unequal. This inequality is strongest for those factors that are most strongly associated with one group or the other. Thus, one way to find these factors and make the distribution more equal through matching is to build a prediction model for the treatment and select the most important factors in the model, which can then be used to train the PS model.

Thus, in our study, to derive robust estimate of the propensity scores, we first selected the most relevant variables that predicts the treatment (head-to-head OAC groups) using the Boruta algorithm.^11^

The Boruta algorithm randomly shuffling each variable in the data and then trains a RF model on the original and permuted variables (also called shadow variables). This process is repeated several times, and on each run the variable importance score (output from the RF model) of each original variable is compared with a threshold. The threshold is the highest importance score recorded among the shadow variables. When the importance of a variable is higher than this threshold, the variable is declared as important and is accepted otherwise the variable is rejected. The idea is that a variable is useful only if it is capable of doing better than the best randomized version of the variable.

The selected variables are then used to build a RF propensity score model. The RF propensity score model also outputs a proximity matrix, which defines how treatment and control patients are similar (see below).

*Proximity matrix*

Other than predicting the response from a set of predictors, an important side-effect of RF is that it outputs a “proximity” matrix, which is a similarity matrix of size $n\times n$, where $n$ is the number of observations. The similarity matrix defines how observations in the data are similar. Specifically, the entrees in the matrix constitute the fraction of times in which two observations are placed in the same terminal node of a tree. The intuition of proximity/similarity is that, if two observations end up in the same terminal node, then they are naturally similar and their proximity score is increased by one. This is done for all observations and trees in the forest and the proximities are normalized by dividing by the number of trees. The proximity matrix can be easily transformed into a dissimilarity matrix: if $s_{ij}\in[0,1]$ is the proximity score of the $i$ and $j$ observations, then the distance between them is given by $d_{ij}=\sqrt{1-s_{ij}}$. The dissimilarity matrix can then be used for unsupervised learning such as clustering or for matching case and control cases in observational data as in this study.

*Matching*

To obtain optimal match pairs, for each treated patient, all control patients with propensity score within a small range (0.2 times the standard deviation of the propensity score for all patients) were selected. Among these control patients, the patient(s) with the smallest distance (highest proximity score) from the treated patient was then selected.

1. A hybrid causal machine learning algorithm

We develop a novel hybrid causal machine learning method based on integrating the causal tree (CT) and the targeted maximum likelihood estimation (TMLE) methods to identify patient subgroups characterizing the treatment heterogeneity of OACs on outcomes.

*Causal Tree*

The CT algorithm is tree-based recursive partitioning algorithm designed to discover patient subgroups with heterogeneous treatment effects from observational data.^12,13^ The algorithm leverages a modification of the decision tree learning algorithm and splits observational data by values of confounding variables in order to optimally estimate heterogeneous treatment effects. In effect, it partitions the training data into clusters such that it maximizes treatment heterogeneity between clusters while minimizing treatment heterogeneity within the clusters. To generate unbiased estimates of the treatment effects, CT separates the decision tree construction step (i.e., partitioning the covariates and pruning the tree) and estimation of the treatment effects. That is, internally the training data are divided into two parts, one part used for growing and pruning the tree and the second part used to estimate treatment effects within the nodes of the tree. This strategy by CT to estimate unbiased and generalizable estimate of heterogeneous treatment effect is known as honest estimation.^12^

*Targeted maximum likelihood estimation*

TMLE is an efficient, double robust, semi-parametric approach for estimating treatment effects that accounts for cofounding in observational data with bias reduction properties.^14-16^ Double robust means that the estimated treatment effect will remain consistent if either the propensity score model or the outcome model are misspecified but the other is properly specified. The outcome model is a model that estimates the probability of an endpoint given the treatment and covariates.

Because our study data was right-censored, the outcome model is a survival model that predicts the survival probability of an endpoint.

A major advantage of the TMLE method is that it can flexibly incorporate a variety of machine learning algorithms for estimating the propensity and outcome models. Thus, to avoid or minimize potential misspecification of the propensity and outcome models, we employed the random forest algorithm to estimate the propensity scores (as described above) and the random survival forest algorithm to estimate the survival probabilities of the endpoints. The random survival forest is an extension of the random forest algorithm for right-censored survival data.^17^

1. Training and validation

Our choice of clustering algorithm (CT), which generates clusters by minimizing treatment heterogeneity for patients within clusters while maximizing between clusters, is uniquely suited to identify similar subgroups of AF patients that may benefit differently from the population-level average treatment effects. However, proper training of the CT algorithm and adequate sample sizes within the nodes of the CT are required to generate stable clusters and consistent estimates of treatment effects.

As such, the maximum depth (*max_depth*) for growing the tree was set to 6, the minimum number of treated or control observations (*n_min_*) in a terminal node was to set 100, and the minimum number of events (*e_min_*) was set to 8. We repeated the training-validation split procedure 1000 times and selected the optimal CT structure based on the best area under the decision curve (AUDC).

1. Diagnosis codes for outcomes

Table 1: Diagnosis Codes for the outcomes

| **Diagnosis Codes** | | | |
| --- | --- | --- | --- |
| **ICD-9-CM** | | **ICD-10-CM** | |
| **Intracranial bleeding** | 430, 431, 432.x, 852.x, 853.x, 800.2x, 800.3x, 800.7x, 800.8x, 801.2x, 801.3x, 801.7x, 801.8x, 803.2x, 803.3x, 803.7x, 803.8x, 804.2x, 804.3x, 804.7x, 804.8x, | | I60.x, I61.x, I69.0x*, I69.1x*, I69.2x*,S06.34x, S06.35x, S06.36x, S06.37x, S06.38x, S06.4x, S06.5x, S06.6x |
| **Gastrointestinal bleeding** | 456.0, 456.20, 530.21, 530.7, 530.82, 531.0x, 531.2x, 531.4x, 531.6x, 532.0x, 532.2x, 532.4x, 532.6x, 533.0x, 533.2x, 533.4x, 533.6x, 534.0x, 534.2x, 534.4x, 534.6x, 535.01, 535.11, 535.21, 535.31, 535.41, 535.51, 535.61, 535.71, 537.83, 537.84, 562.02, 562.03, 562.12, 562.13, 569.3, 569.85, 578.x | | I85.01, I85.11, K22.11,K22.6, K25.0, K25.2, K25.4, K25.6, K26.0, K26.2, K26.4, K26.6 ,K27.0, K27.2, K27.4, K27.6, K28.0, K28.4, K28.6, K29.x1, K31.811, K31.82, K55.21, K57.x1, K57.x3, K62.5, K63.81, , K92.0, K92.1, K92.2, |
| **Other bleeding** | 423.0, 459.0, 568.81, 596.7, 599.71, 719.1x, 784.8, 786.3 | | I31.2, K66.1, M25.0, R04.1, R04.2, R31.0, R58 |
| **Hemorrhagic stroke** | 430, 431 | | I60.x, I61.x, I69.0x*, I69.1x* |
| **Ischemic stroke** | 433.x1, 434.x1, 436 | | I63.x, I69.3x* |
| **Systemic embolism** | 444.x | | I74.x |
| *these codes were used when identifying medical history, not new events.  Note. We did not present diagnosis codes for comorbidities due to a large number of codes. The SAS codes for all the comorbidities is available upon request. | | | |

1. Supplementary Results

Table 2: Baseline characteristics by head-to-head OAC treatment groups after matching

| **Variables** | **Apixaban**  **vs**  **Dabigatran** | **Apixaban**  **vs**  **Rivaroxaban** | **Dabigatran**  **vs**  **Rivaroxaban** | **Apixaban**  **vs**  **Warfarin** | **Dabigatran**  **vs**  **Warfarin** | **Rivaroxaban**  **vs**  **Warfarin** |
| --- | --- | --- | --- | --- | --- | --- |
| n | 5949 | 12827 | 11451 | 17373 | 13929 | 16403 |
| Age | 67.74 (10.90) | 69.62 (11.00) | 68.98 (11.07) | 72.77 (9.83) | 72.03 (10.13) | 72.40 (9.95) |
| **eGFR groups** | | | | | | |
| 15-30 | 81 ( 1.4) | 198 ( 1.5) | 141 ( 1.2) | 790 ( 4.5) | 619 ( 4.4) | 682 ( 4.2) |
| 30-45 | 389 ( 6.5) | 1001 ( 7.8) | 789 ( 6.9) | 2094 (12.1) | 1596 (11.5) | 1873 (11.4) |
| 45-60 | 1027 (17.3) | 2416 (18.8) | 2011 (17.6) | 3573 (20.6) | 2833 (20.3) | 3360 (20.5) |
| 60-90 | 3240 (54.5) | 6854 (53.4) | 6348 (55.4) | 8739 (50.3) | 6974 (50.1) | 8338 (50.8) |
| 90+ | 1212 (20.4) | 2358 (18.4) | 2162 (18.9) | 2177 (12.5) | 1907 (13.7) | 2150 (13.1) |
| **CHADS2VASC** | | | | | | |
| 0 | 176 ( 3.0) | 303 ( 2.4) | 301 ( 2.6) | 155 ( 0.9) | 157 ( 1.1) | 166 ( 1.0) |
| 1 | 536 ( 9.0) | 943 ( 7.4) | 908 ( 7.9) | 589 ( 3.4) | 524 ( 3.8) | 589 ( 3.6) |
| 2 | 875 (14.7) | 1708 (13.3) | 1579 (13.8) | 1253 ( 7.2) | 1113 ( 8.0) | 1261 ( 7.7) |
| 3+ | 4362 (73.3) | 9873 (77.0) | 8663 (75.7) | 15376 (88.5) | 12135 (87.1) | 14387 (87.7) |
| **HASBLED** | | | | | | |
| 0 | 209 ( 3.5) | 338 ( 2.6) | 335 ( 2.9) | 215 ( 1.2) | 199 ( 1.4) | 210 ( 1.3) |
| 1 | 906 (15.2) | 1603 (12.5) | 1536 (13.4) | 1174 ( 6.8) | 1061 ( 7.6) | 1226 ( 7.5) |
| 2 | 1629 (27.4) | 3363 (26.2) | 3141 (27.4) | 3736 (21.5) | 3094 (22.2) | 3655 (22.3) |
| 3 | 1699 (28.6) | 3701 (28.9) | 3347 (29.2) | 5363 (30.9) | 4308 (30.9) | 5111 (31.2) |
| 4 | 1014 (17.0) | 2511 (19.6) | 2058 (18.0) | 4292 (24.7) | 3313 (23.8) | 3906 (23.8) |
| 5 | 408 ( 6.9) | 1041 ( 8.1) | 836 ( 7.3) | 1991 (11.5) | 1515 (10.9) | 1773 (10.8) |
| 6 | 71 ( 1.2) | 231 ( 1.8) | 168 ( 1.5) | 522 ( 3.0) | 377 ( 2.7) | 450 ( 2.7) |
| 7 | 11 ( 0.2) | 37 ( 0.3) | 28 ( 0.2) | 77 ( 0.4) | 60 ( 0.4) | 70 ( 0.4) |
| **US Region** | | | | | | |
| Midwest | 802 (13.5) | 2013 (15.7) | 1649 (14.4) | 3260 (18.8) | 2543 (18.3) | 2990 (18.2) |
| Northeast | 945 (15.9) | 2232 (17.4) | 2068 (18.1) | 3035 (17.5) | 2455 (17.6) | 2999 (18.3) |
| South | 3584 (60.2) | 7292 (56.8) | 6571 (57.4) | 9410 (54.2) | 7537 (54.1) | 8794 (53.6) |
| West | 618 (10.4) | 1290 (10.1) | 1163 (10.2) | 1668 ( 9.6) | 1394 (10.0) | 1620 ( 9.9) |
| **Race** | | | | | | |
| Asian | 147 ( 2.5) | 356 ( 2.8) | 355 ( 3.1) | 352 ( 2.0) | 317 ( 2.3) | 379 ( 2.3) |
| Black | 514 ( 8.6) | 1207 ( 9.4) | 946 ( 8.3) | 1896 (10.9) | 1475 (10.6) | 1682 (10.3) |
| Hispanic | 352 ( 5.9) | 904 ( 7.0) | 792 ( 6.9) | 1078 ( 6.2) | 884 ( 6.3) | 1047 ( 6.4) |
| White | 4441 (74.7) | 9241 (72.0) | 8457 (73.9) | 12711 (73.2) | 10231 (73.5) | 12104 (73.8) |
| Other | 495 ( 8.3) | 1119 ( 8.7) | 901 ( 7.9) | 1336 ( 7.7) | 1022 ( 7.3) | 1191 ( 7.3) |
| Gender: female | 2240 (37.7) | 5288 (41.2) | 4486 (39.2) | 7654 (44.1) | 5911 (42.4) | 7082 (43.2) |
| **Comorbidities/Conditions/Procedures** | | | | | | |
| HF | 1783 (30.0) | 4123 (32.1) | 3497 (30.5) | 7808 (44.9) | 6117 (43.9) | 7183 (43.8) |
| HTN | 5395 (90.7) | 11754 (91.6) | 10473 (91.5) | 16461 (94.8) | 13148 (94.4) | 15533 (94.7) |
| Thrombo | 1091 (18.3) | 2476 (19.3) | 2049 (17.9) | 4457 (25.7) | 3541 (25.4) | 4112 (25.1) |
| Diabetes | 2353 (39.6) | 5207 (40.6) | 4562 (39.8) | 7996 (46.0) | 6436 (46.2) | 7510 (45.8) |
| CAD | 3146 (52.9) | 6703 (52.3) | 5905 (51.6) | 10697 (61.6) | 8566 (61.5) | 9990 (60.9) |
| PAD | 680 (11.4) | 1647 (12.8) | 1399 (12.2) | 3125 (18.0) | 2484 (17.8) | 2873 (17.5) |
| HMB | 1378 (23.2) | 2922 (22.8) | 2596 (22.7) | 4606 (26.5) | 3704 (26.6) | 4275 (26.1) |
| HIB | 114 ( 1.9) | 224 ( 1.7) | 186 ( 1.6) | 425 ( 2.4) | 336 ( 2.4) | 357 ( 2.2) |
| Liver Disease | 913 (15.3) | 2028 (15.8) | 1773 (15.5) | 2624 (15.1) | 2122 (15.2) | 2478 (15.1) |
| Alcoholism | 326 ( 5.5) | 773 ( 6.0) | 669 ( 5.8) | 948 ( 5.5) | 773 ( 5.5) | 893 ( 5.4) |
| Obesity | 2006 (33.7) | 4797 (37.4) | 4038 (35.3) | 5761 (33.2) | 4492 (32.2) | 5357 (32.7) |
| Smoke | 1975 (33.2) | 4514 (35.2) | 3837 (33.5) | 6115 (35.2) | 4767 (34.2) | 5709 (34.8) |
| Falls | 702 (11.8) | 1801 (14.0) | 1416 (12.4) | 2818 (16.2) | 2131 (15.3) | 2572 (15.7) |
| AKI | 677 (11.4) | 1854 (14.5) | 1408 (12.3) | 4009 (23.1) | 3086 (22.2) | 3605 (22.0) |
| VHD | 2876 (48.3) | 6151 (48.0) | 5492 (48.0) | 9016 (51.9) | 7285 (52.3) | 8495 (51.8) |
| Non skin cancer | 1064 (17.9) | 2399 (18.7) | 2099 (18.3) | 3856 (22.2) | 3066 (22.0) | 3565 (21.7) |
| Recent major bleed | 28 ( 0.5) | 59 ( 0.5) | 48 ( 0.4) | 135 ( 0.8) | 127 ( 0.9) | 126 ( 0.8) |
| Recent Thrombo | 253 ( 4.3) | 558 ( 4.4) | 437 ( 3.8) | 1185 ( 6.8) | 975 ( 7.0) | 1116 ( 6.8) |
| Hyperlipidemia | 5345 (89.8) | 11333 (88.4) | 10187 (89.0) | 15715 (90.5) | 12638 (90.7) | 14873 (90.7) |
| Ischemic Stroke | 747 (12.6) | 1680 (13.1) | 1365 (11.9) | 3258 (18.8) | 2530 (18.2) | 2949 (18.0) |
| MI | 928 (15.6) | 2109 (16.4) | 1812 (15.8) | 4011 (23.1) | 3166 (22.7) | 3725 (22.7) |
| COPD | 793 (13.3) | 1895 (14.8) | 1679 (14.7) | 3282 (18.9) | 2636 (18.9) | 3114 (19.0) |
| Obs Sleep Apnea | 1510 (25.4) | 3159 (24.6) | 2749 (24.0) | 3643 (21.0) | 2956 (21.2) | 3540 (21.6) |
| Systolic HF | 647 (10.9) | 1668 (13.0) | 1305 (11.4) | 3264 (18.8) | 2446 (17.6) | 2905 (17.7) |
| Cardioversion | 856 (14.4) | 1758 (13.7) | 1525 (13.3) | 1529 ( 8.8) | 1249 ( 9.0) | 1421 ( 8.7) |
| Ablation | 249 ( 4.2) | 420 ( 3.3) | 407 ( 3.6) | 333 ( 1.9) | 304 ( 2.2) | 326 ( 2.0) |
| Pacemaker/ICD | 667 (11.2) | 1560 (12.2) | 1306 (11.4) | 2529 (14.6) | 1940 (13.9) | 2232 (13.6) |
| PCI/CABG | 1013 (17.0) | 2120 (16.5) | 1831 (16.0) | 4137 (23.8) | 3289 (23.6) | 3802 (23.2) |
| Depression | 2195 (36.9) | 4837 (37.7) | 4148 (36.2) | 6854 (39.5) | 5347 (38.4) | 6336 (38.6) |
| Dementia | 315 ( 5.3) | 792 ( 6.2) | 623 ( 5.4) | 1432 ( 8.2) | 1051 ( 7.5) | 1252 ( 7.6) |
| Hypothyroidism | 1865 (31.3) | 4048 (31.6) | 3604 (31.5) | 5910 (34.0) | 4720 (33.9) | 5537 (33.8) |
| Thyrotoxicosis | 341 ( 5.7) | 669 ( 5.2) | 632 ( 5.5) | 928 ( 5.3) | 783 ( 5.6) | 893 ( 5.4) |
| Ulcer | 345 ( 5.8) | 739 ( 5.8) | 644 ( 5.6) | 1173 ( 6.8) | 945 ( 6.8) | 1090 ( 6.6) |
| **Medications** | | | | | | |
| Antiplatelet | 637 (10.7) | 1463 (11.4) | 1215 (10.6) | 2431 (14.0) | 1896 (13.6) | 2198 (13.4) |
| NSAIDS | 735 (12.4) | 1585 (12.4) | 1405 (12.3) | 1929 (11.1) | 1555 (11.2) | 1782 (10.9) |
| Amiodarone | 663 (11.1) | 1384 (10.8) | 1154 (10.1) | 2134 (12.3) | 1718 (12.3) | 1970 (12.0) |
| Dronedarone | 253 ( 4.3) | 380 ( 3.0) | 376 ( 3.3) | 297 ( 1.7) | 284 ( 2.0) | 305 ( 1.9) |
| OAAD | 808 (13.6) | 1534 (12.0) | 1434 (12.5) | 1294 ( 7.4) | 1074 ( 7.7) | 1230 ( 7.5) |
| Digoxin | 613 (10.3) | 954 ( 7.4) | 977 ( 8.5) | 1884 (10.8) | 1649 (11.8) | 1801 (11.0) |
| Diltiazem | 1079 (18.1) | 2293 (17.9) | 2053 (17.9) | 3082 (17.7) | 2451 (17.6) | 2859 (17.4) |
| Verapamil | 106 ( 1.8) | 225 ( 1.8) | 211 ( 1.8) | 308 ( 1.8) | 288 ( 2.1) | 327 ( 2.0) |
| ORC | 3834 (64.4) | 8382 (65.3) | 7326 (64.0) | 11633 (67.0) | 9195 (66.0) | 10838 (66.1) |
| ACE/ARB | 3157 (53.1) | 6807 (53.1) | 6003 (52.4) | 9532 (54.9) | 7569 (54.3) | 8880 (54.1) |
| CCB | 1263 (21.2) | 2835 (22.1) | 2487 (21.7) | 4128 (23.8) | 3264 (23.4) | 3837 (23.4) |
| Obeta | 299 ( 5.0) | 661 ( 5.2) | 587 ( 5.1) | 1109 ( 6.4) | 950 ( 6.8) | 1065 ( 6.5) |
| Loop Diuretics | 1151 (19.3) | 2804 (21.9) | 2268 (19.8) | 5040 (29.0) | 3966 (28.5) | 4658 (28.4) |
| Thiaziades | 1409 (23.7) | 2942 (22.9) | 2675 (23.4) | 3965 (22.8) | 3250 (23.3) | 3766 (23.0) |
| Statins | 3027 (50.9) | 6483 (50.5) | 5673 (49.5) | 9407 (54.1) | 7328 (52.6) | 8641 (52.7) |
| Insulin | 427 ( 7.2) | 943 ( 7.4) | 758 ( 6.6) | 1662 ( 9.6) | 1339 ( 9.6) | 1531 ( 9.3) |
| Metformin | 933 (15.7) | 2048 (16.0) | 1804 (15.8) | 2643 (15.2) | 2113 (15.2) | 2511 (15.3) |
| Odiab | 746 (12.5) | 1663 (13.0) | 1411 (12.3) | 2451 (14.1) | 1943 (13.9) | 2263 (13.8) |
| Antiulcer agents | 1433 (24.1) | 3219 (25.1) | 2666 (23.3) | 4973 (28.6) | 3875 (27.8) | 4512 (27.5) |
| **Outcomes** | | | | | | |
| Major Bleeding | 102 ( 1.7) | 326 ( 2.5) | 306 ( 2.7) | 644 ( 3.7) | 577 ( 4.1) | 694 ( 4.2) |
| All-cause Mortality | 213 ( 3.6) | 447 ( 3.5) | 433 ( 3.8) | 1067 ( 6.1) | 992 ( 7.1) | 1080 ( 6.6) |
| Primary composite | 277 ( 4.7) | 557 ( 4.3) | 544 ( 4.8) | 1332 ( 7.7) | 1241 ( 8.9) | 1349 ( 8.2) |
| **Follow up time (years)** | | | | | | |
| Follow up Major Bleeding | 0.68 (0.80) | 0.67 (0.72) | 0.69 (0.79) | 0.67 (0.74) | 0.69 (0.81) | 0.68 (0.77) |
| Follow up All-cause Mortality | 0.73 (0.87) | 0.70 (0.75) | 0.74 (0.84) | 0.74 (0.82) | 0.78 (0.92) | 0.76 (0.86) |
| Follow up Primary Composite | 0.68 (0.80) | 0.68 (0.72) | 0.70 (0.79) | 0.68 (0.75) | 0.70 (0.82) | 0.69 (0.78) |
| Unless otherwise noted, data are presented as n (%) for categorical variables and mean (SD) for continuous variables. CABG indicates coronary artery bypass graft; COPD, chronic obstructive pulmonary disease; eGFR, glomerular filtration rate; GI, gastrointestinal; ICD, implantable cardioverter-defibrillator; NSAID, nonsteroidal anti-inflammatory drug; PCI, percutaneous coronary intervention.  *The CHADS2VASC score ranges from 0 to 9; higher score indicates higher risk of stroke. A point score is calculated as 1 point each for heart failure, hypertension, diabetes mellitus, vascular disease, age 65 to 74 years, and female sex; 2 points for age ≥75 y and prior stroke, TIA, or thromboembolism.  †The HASBLED score ranges from 0 to 9; higher score indicates higher risk of bleeding. A point score is calculated as 1 point each for hypertension, abnormal kidney function, abnormal liver function, prior stroke, prior bleeding or bleeding predisposition, labile international normalized ratio (INR), older than 65 years, medication usage predisposing to bleeding, and alcohol use. This study did not consider INR, so the range of HASBLED was 0–8. | | | | | | |

Table 3: Population ATE. Population level treatment effect of OACs on the primary composite outcome. All values were calculated using matched head-to-head OAC comparison groups.

| **Treatment Groups** | **Outcome** | **n** | **Max follow up (years)** | **ATE** |
| --- | --- | --- | --- | --- |
| Apixaban  vs  Dabigatran | Major Bleeding | 5949 | 6.91 | -0.007(-0.013,-0.002) |
|  | All-cause Mortality |  |  | -0.020(-0.028,-0.011) |
|  | Composite |  |  | -0.023(-0.032,-0.014) |
| Apixaban  vs  Rivaroxaban | Major Bleeding | 12827 | 5.49 | -0.011(-0.015,-0.006) |
|  | All-cause Mortality |  |  | -0.007(-0.012,-0.001) |
|  | Composite |  |  | -0.007(-0.013,-0.001) |
| Dabigatran  vs  Rivaroxaban | Major Bleeding | 11451 | 6.54 | -0.005(-0.01,0.00) |
|  | All-cause Mortality |  |  | 0.012(0.005,0.019) |
|  | Composite |  |  | 0.016(0.008,0.023) |
| Apixaban  vs  Warfarin | Major Bleeding | 17373 | 6.28 | -0.023(-0.027,-0.018) |
|  | All-cause Mortality |  | 6.32 | -0.034(-0.04,-0.028) |
|  | Composite |  | 6.6 | -0.043(-0.049,-0.037) |
| Dabigatran  vs  Warfarin | Major Bleeding | 13929 | 6.93 | -0.012(-0.018,-0.005) |
|  | All-cause Mortality |  | 6.93 | -0.002(-0.012,0.008) |
|  | Composite |  | 6.91 | 0.004(-0.009,0.017) |
| Rivaroxaban  vs  Warfarin | Major Bleeding | 16403 | 6.69 | -0.006(-0.012,-0.001) |
|  | All-cause Mortality |  | 6.79 | -0.022(-0.029,-0.016) |
|  | Composite |  | 6.31 | -0.028(-0.035,-0.021) |

Figure 2: Subgroups of apixaban vs dabigatran users with respect to major bleeding. The subgroups are the terminal nodes of the optimal causal ML model. The green subgroups favor the use of apixaban. All the values were estimated based on the matched sample of apixaban and dabigatran users. ATE indicates average treatment effect; and ERPO, events rate per 1000.

Figure 3: Subgroups of apixaban vs rivaroxaban users with respect to major bleeding. The subgroups are the terminal nodes of the optimal causal ML model. The green subgroups favor the use of apixaban. All the values were estimated based on the matched sample of apixaban and dabigatran users. ATE indicates average treatment effect; and ERPO, events rate per 1000.

Figure 4: Subgroups of dabigatran vs rivaroxaban users with respect to major bleeding. The subgroups are the terminal nodes of the optimal causal ML model. The green subgroups favor the use of dabigatran. All the values were estimated based on the matched sample of apixaban and dabigatran users. ATE indicates average treatment effect; and ERPO, events rate per 1000.

Figure 5: Subgroups of apixaban vs warfarin users with respect to major bleeding. The subgroups are the terminal nodes of the optimal causal ML model. The green subgroups favor the use of apixaban. All the values were estimated based on the matched sample of apixaban and dabigatran users. ATE indicates average treatment effect; and ERPO, events rate per 1000.

Figure 6: Subgroups of dabigatran vs warfarin users with respect to major bleeding. The subgroups are the terminal nodes of the optimal causal ML model. The green subgroup favor the use of dabigatran. All the values were estimated based on the matched sample of apixaban and dabigatran users. ATE indicates average treatment effect; and ERPO, events rate per 1000.

Figure 7: Subgroups of rivaroxaban vs warfarin users with respect to major bleeding. The subgroups are the terminal nodes of the optimal causal ML model. The green subgroups favor the use of rivaroxaban. All the values were estimated based on the matched sample of apixaban and dabigatran users. ATE indicates average treatment effect; and ERPO, events rate per 1000.

Figure 8: Subgroups of apixaban vs dabigatran users with respect to all-cause mortality. The subgroups are the terminal nodes of the optimal causal ML model. The green subgroups favor the use of apixaban. All the values were estimated based on the matched sample of apixaban and dabigatran users. ATE indicates average treatment effect; and ERPO, events rate per 1000.

Figure 9: Subgroups of apixaban vs rivaroxaban users with respect to all-cause mortality. The subgroups are the terminal nodes of the optimal causal ML model. The green subgroups favor the use of apixaban. All the values were estimated based on the matched sample of apixaban and dabigatran users. ATE indicates average treatment effect; and ERPO, events rate per 1000.

Figure 10: Subgroups of dabigatran vs rivaroxaban users with respect to all-cause mortality. The subgroups are the terminal nodes of the optimal causal ML model. The green subgroup favor the use of dabigatran, red subgroups favor rivaroxaban. All the values were estimated based on the matched sample of apixaban and dabigatran users. ATE indicates average treatment effect; and ERPO, events rate per 1000.

Figure 11: Subgroups of apixaban vs warfarin users with respect to all-cause mortality. The subgroups are the terminal nodes of the optimal causal ML model. The green subgroups favor the use of warfarin. All the values were estimated based on the matched sample of apixaban and dabigatran users. ATE indicates average treatment effect; and ERPO, events rate per 1000.

Figure 12: Subgroups of dabigatran vs warfarin users with respect to all-cause mortality. The subgroups are the terminal nodes of the optimal causal ML model. The green subgroups favor the use of dabigatran, red subgroups favor warfarin. All the values were estimated based on the matched sample of apixaban and dabigatran users. ATE indicates average treatment effect; and ERPO, events rate per 1000.

Figure 13: Subgroups of rivaroxaban vs warfarin users with respect to all-cause mortality. The subgroups are the terminal nodes of the optimal causal ML model. The green subgroups favor the use of rivaroxaban. All the values were estimated based on the matched sample of apixaban and dabigatran users. ATE indicates average treatment effect; and ERPO, events rate per 1000.

Table 4: Subgroup treatment effects of OACs on the primary composite outcome. K represents the subgroup number. The coloring indicate subgroups for which the ATE is significant, i.e., the 95 % confidence interval (CI) does not include 0. In the comparison of drug A vs drug B, red indicate subgroups favoring drug A, green indicate subgroups favoring drug B, while black indicate subgroups favoring neither drug. All numbers were calculated using matched head-to-head OAC comparison groups. Event rates was calculated as number of events per 1000 person-year.

| **Treatment Groups** | **K** | **Size** | **Max Follow up (yrs)** | **No Events (Event Rates)** | **ATE (95% CI)** | **Key patient factors** |
| --- | --- | --- | --- | --- | --- | --- |
| Apixaban vs Dabigatran | 1 | 338 | 6.8 | 47(153.36) | -0.021(-0.081,0.039) | Age ≥ 80.5 & Age < 82.5 |
|  | 2 | 468 | 4.7 | 42(142.53) | -0.079(-0.122,-0.037) | Age ≥ 82.5 |
|  | 3 | 425 | 5.3 | 9(36.39) | -0.028(-0.05,-0.006) | Age < 80.5 & Race = Other |
|  | 4 | 3884 | 6.9 | 99(37.18) | -0.009(-0.017,0) | Age < 80.5 & Race = Asian, Black, Hispanic, White & Prescribed Loop Diuretics = No |
|  | 5 | 525 | 4.6 | 61(185.82) | -0.054(-0.102,-0.006) | Age < 80.5 & Race = Asian, Black, Hispanic, White & Prescribed Loop Diuretics = Yes & Obs.Sleep.Apnea =No |
|  | 6 | 309 | 4.8 | 19(88.13) | -0.063(-0.107,-0.019) | Age < 80.5 & Race = Asian, Black, Hispanic, White & Prescribed Loop Diuretics = Yes & Obs.Sleep.Apnea =Yes |
| Apixaban vs Rivaroxaban | 1 | 625 | 5 | 52(112.13) | -0.008(-0.044,0.029) | Ischemic Stroke =Yes & HASBLED = 4, 5, 6, 7, 8 & AKI =No & Obesity =No |
|  | 2 | 300 | 3.8 | 18(86.24) | -0.025(-0.068,0.018) | Ischemic Stroke =Yes & HASBLED = 4, 5, 6, 7, 8 & AKI =No & Obesity =Yes |
|  | 3 | 383 | 4.5 | 47(227.41) | -0.035(-0.09,0.02) | Ischemic Stroke =Yes & HASBLED = 4, 5, 6, 7, 8 & AKI =Yes |
|  | 4 | 372 | 5.1 | 26(95.39) | -0.055(-0.094,-0.017) | Ischemic Stroke =Yes & HASBLED = 1, 2, 3 |
|  | 5 | 492 | 4.1 | 38(131.14) | 0.024(-0.018,0.066) | Ischemic Stroke =No & Antiulcer =Yes & AKI =Yes |
|  | 6 | 575 | 4.7 | 14(38.85) | -0.020(-0.039,-0.002) | Ischemic Stroke =No & Antiulcer =Yes & AKI =No & Age < 64 |
|  | 7 | 1617 | 4.8 | 88(76.42) | -0.002(-0.022,0.018) | Ischemic Stroke =No & Antiulcer =Yes & AKI =No & Age ≥ 64 |
|  | 8 | 6407 | 5.4 | 186(41.38) | -0.004(-0.012,0.003) | Ischemic Stroke =No & Antiulcer =No & Systolic.HF =No & CHADS2VASC = 2, 3+ |
|  | 9 | 1056 | 5.5 | 9(15.05) | -0.005(-0.013,0.003) | Ischemic Stroke =No & Antiulcer =No & Systolic.HF =No & CHADS2VASC = 0, 1 |
|  | 10 | 364 | 3.9 | 16(64.67) | 0.000(-0.036,0.036) | Ischemic Stroke =No & Antiulcer =No & Systolic.HF =Yes & HASBLED = 0, 1, 2 |
|  | 11 | 636 | 4.1 | 63(171.99) | -0.020(-0.06,0.02) | Ischemic Stroke =No & Antiulcer =No & Systolic.HF =Yes & HASBLED = 3, 4, 5, 6, 7 |
| Dabigatran vs Rivaroxaban | 1 | 611 | 4.5 | 25(61.37) | 0.010(-0.021,0.042) | eGFR = 90+ & Region = Midwest, Northeast |
|  | 2 | 500 | 5.5 | 12(37.69) | -0.006(-0.029,0.018) | eGFR = 90+ & Region = South, West & Obs.Sleep.Apnea =Yes |
|  | 3 | 658 | 5.7 | 19(43.88) | -0.001(-0.019,0.017) | eGFR = 90+ & Region = South, West & Obs.Sleep.Apnea =No & ORC =Yes |
|  | 4 | 393 | 5.2 | 8(33.35) | -0.019(-0.039,0.001) | eGFR = 90+ & Region = South, West & Obs.Sleep.Apnea =No & ORC =No |
|  | 5 | 1174 | 6.5 | 23(29.2) | 0.015(-0.002,0.033) | eGFR = 15-30, 30-45, 45-60, 60-90 & HASBLED = 0, 1 |
|  | 6 | 490 | 4.4 | 12(38.1) | -0.023(-0.043,-0.003) | eGFR = 15-30, 30-45, 45-60, 60-90 & HASBLED = 2, 3, 4, 5, 6, 7, 8 & Age ≥ 64 & Age < 66.5 |
|  | 7 | 6499 | 6.9 | 406(85.65) | 0.027(0.015,0.038) | eGFR = 15-30, 30-45, 45-60, 60-90 & HASBLED = 2, 3, 4, 5, 6, 7, 8 & Age ≥ 64 & Age ≥ 66.5 |
|  | 8 | 525 | 4.8 | 20(61.44) | 0.008(-0.024,0.04) | eGFR = 15-30, 30-45, 45-60, 60-90 & HASBLED = 2, 3, 4, 5, 6, 7, 8 & Age < 64 & VHD =Yes |
|  | 9 | 601 | 6.4 | 19(43.68) | 0.008(-0.014,0.03) | eGFR = 15-30, 30-45, 45-60, 60-90 & HASBLED = 2, 3, 4, 5, 6, 7, 8 & Age < 64 & VHD =No |
| Apixaban vs Warfarin | 1 | 403 | 6.3 | 39(126.09) | -0.060(-0.106,-0.015) | Thrombo =Yes & Depression =Yes & Region = Midwest |
|  | 2 | 1412 | 5.8 | 166(189.66) | -0.073(-0.099,-0.048) | Thrombo =Yes & Depression =Yes & Region = Northeast, South, West & CAD =Yes |
|  | 3 | 445 | 5.3 | 32(104.21) | -0.063(-0.101,-0.025) | Thrombo =Yes & Depression =Yes & Region = Northeast, South, West & CAD =No |
|  | 4 | 479 | 4.4 | 59(188.91) | -0.083(-0.126,-0.039) | Thrombo =Yes & Depression =No & Race = Asian, Black, Hispanic |
|  | 5 | 795 | 5.1 | 87(160.66) | -0.043(-0.079,-0.008) | Thrombo =Yes & Depression =No & Race = White, Other & Diabetes =Yes |
|  | 6 | 923 | 4.6 | 61(83.56) | -0.031(-0.059,-0.004) | Thrombo =Yes & Depression =No & Race = White, Other & Diabetes =No |
|  | 7 | 6632 | 6.9 | 400(82.63) | -0.033(-0.042,-0.023) | Thrombo =No & Age ≥ 64 & eGFR = 45-60, 60-90, 90+ & Non.skin.cancer =No |
|  | 8 | 1905 | 6.7 | 158(124.18) | -0.054(-0.073,-0.035) | Thrombo =No & Age ≥ 64 & eGFR = 45-60, 60-90, 90+ & Non.skin.cancer =Yes |
|  | 9 | 1867 | 5.9 | 235(196.77) | -0.053(-0.078,-0.027) | Thrombo =No & Age ≥ 64 & eGFR = 15-30, 30-45 |
|  | 10 | 412 | 3.9 | 34(150.8) | -0.046(-0.087,-0.006) | Thrombo =No & Age < 64 & AKI =Yes |
|  | 11 | 1687 | 5.8 | 23(25.57) | -0.019(-0.028,-0.01) | Thrombo =No & Age < 64 & AKI =No & Prescribed Loop Diuretics =No |
|  | 12 | 413 | 3.7 | 38(149.98) | -0.037(-0.082,0.007) | Thrombo =No & Age < 64 & AKI =No & Prescribed Loop Diuretics =Yes |
| Dabigatran vs Warfarin | 1 | 826 | 6.9 | 143(241.71) | 0.098(0.039,0.157) | Race = Asian, Black & HASBLED = 4, 5, 6, 7 |
|  | 2 | 966 | 5.4 | 96(143.17) | -0.023(-0.058,0.013) | Race = Asian, Black & HASBLED = 0, 1, 2, 3 |
|  | 3 | 711 | 5.4 | 28(59.35) | -0.005(-0.028,0.019) | Race = Hispanic, White, Other & HTN =No |
|  | 4 | 7780 | 6.8 | 733(138.08) | 0.012(-0.008,0.032) | Race = Hispanic, White, Other & HTN =Yes & Prescribed Thiaziades =No & CHADS2VASC = 3+ |
|  | 5 | 891 | 6.9 | 12(22.27) | -0.011(-0.025,0.002) | Race = Hispanic, White, Other & HTN =Yes & Prescribed Thiaziades =No & CHADS2VASC = 1, 2 |
|  | 6 | 2219 | 6.7 | 201(112.14) | -0.023(-0.044,-0.003) | Race = Hispanic, White, Other & HTN =Yes & Prescribed Thiaziades =Yes & Age ≥ 64.5 |
|  | 7 | 536 | 6.5 | 28(74.27) | -0.004(-0.034,0.026) | Race = Hispanic, White, Other & HTN =Yes & Prescribed Thiaziades =Yes & Age < 64.5 |
| Rivaroxaban vs Warfarin | 1 | 817 | 5.9 | 85(167.79) | -0.020(-0.057,0.017) | MI =No & Age ≥ 71 & AKI =Yes & eGFR = 45-60, 60-90, 90+ |
|  | 2 | 672 | 5.9 | 93(217.87) | -0.060(-0.101,-0.019) | MI =No & Age ≥ 71 & AKI =Yes & eGFR = 15-30, 30-45 |
|  | 3 | 1812 | 6.7 | 141(103.62) | -0.019(-0.041,0.003) | MI =No & Age ≥ 71 & AKI =No & Smoke =Yes |
|  | 4 | 4319 | 6.9 | 358(103.36) | -0.026(-0.039,-0.012) | MI =No & Age ≥ 71 & AKI =No & Smoke =No |
|  | 5 | 612 | 5.2 | 66(184.97) | -0.071(-0.107,-0.035) | MI =No & Age < 71 & Systolic HF =Yes |
|  | 6 | 531 | 4 | 41(114.78) | 0.004(-0.034,0.042) | MI =No & Age < 71 & Systolic HF =No & COPD =Yes |
|  | 7 | 3915 | 5.9 | 114(44.56) | -0.018(-0.027,-0.01) | MI =No & Age < 71 & Systolic HF =No & COPD =No |
|  | 8 | 701 | 6.5 | 82(178.55) | -0.055(-0.094,-0.016) | MI =Yes & Region = Midwest |
|  | 9 | 734 | 4.4 | 92(210.15) | 0.008(-0.035,0.05) | MI =Yes & Region = Northeast, South, West & Obs.Sleep.Apnea =Yes |
|  | 10 | 1629 | 5.8 | 182(180.97) | -0.036(-0.06,-0.012) | MI =Yes Obs.Sleep.Apnea =No & Region = South, West |
|  | 11 | 661 | 5.1 | 95(216.1) | -0.079(-0.115,-0.042) | MI =Yes & Obs.Sleep.Apnea =No & Region = Northeast |

Table 5: Subgroup treatment effects of OACs on the secondary outcomes. K represents the subgroup number. The coloring indicate subgroups for which the ATE is significant. In the comparison of drug A vs drug B, red indicate subgroups favoring drug A, green indicate subgroups favoring drug B, while black indicate subgroups favoring neither drug. All numbers were calculated using matched head-to-head OAC comparison groups. Event rates was calculated as number of events per 1000 person-year.

| **Outcome** | **Treatment groups** | **K** | **Size** | **Max Follow up (yrs)** | **N0 Events (Rates)** | **ATE (95% CI)** | **Key patient factors** |
| --- | --- | --- | --- | --- | --- | --- | --- |
| Major Bleeding | Apixaban vs Dabigatran | 1 | 1409 | 6.5 | 26(23.46) | -0.011(-0.022,0.001) | Prescribed Thiaziades =Yes |
|  |  | 2 | 3618 | 6.9 | 48(20.37) | -0.005(-0.012,0.001) | Prescribed Thiaziades =No prescribed Loop Diuretics = No |
|  |  | 3 | 522 | 4.6 | 16(45.45) | -0.029(-0.052,-0.006) | Thiaziades =No & Prescribed Loop Diuretics = Yes & Obesity =No |
|  |  | 4 | 400 | 4 | 12(50.88) | 0.015(-0.013,0.042) | Prescribed Thiaziades =No & Prescribed Loop.Diuretics = Yes & Obesity =Yes |
|  | Apixaban vs Rivaroxaban | 1 | 836 | 4.8 | 10(18.93) | -0.013(-0.024,-0.003) | CHADS2VASC = 2, 3+ & Age < 69 & Obesity =No & Age < 62 |
|  |  | 2 | 1228 | 5.2 | 21(24.11) | -0.011(-0.022,0) | CHADS2VASC = 2, 3+ & Age < 69 & Obesity =No & Age ≥ 62 |
|  |  | 3 | 1312 | 5.3 | 20(22.92) | -0.014(-0.024,-0.003) | CHADS2VASC = 2, 3+ & Age < 69 & Obesity =Yes & Diabetes =Yes |
|  |  | 4 | 902 | 4.7 | 12(20.63) | -0.016(-0.027,-0.004) | CHADS2VASC = 2, 3+ & Age < 69 & Obesity =Yes & Diabetes =No |
|  |  | 5 | 916 | 5.1 | 40(58.13) | -0.035(-0.057,-0.012) | CHADS2VASC = 2, 3+ & Age ≥ 69 & Ischemic Stroke =Yes & AKI =No |
|  |  | 6 | 327 | 3.3 | 17(97.44) | -0.043(-0.086,0) | CHADS2VASC = 2, 3+ & Age ≥ 69 & Ischemic Stroke =Yes & AKI =Yes |
|  |  | 7 | 2608 | 4.8 | 71(37.15) | 0.000(-0.012,0.012) | CHADS2VASC = 2, 3+ & Age ≥ 69 & Ischemic Stroke =No & CAD =No |
|  |  | 8 | 3452 | 5.2 | 124(54.5) | -0.014(-0.024,-0.004) | CHADS2VASC = 2, 3+ & Age ≥ 69 & Ischemic Stroke =No & CAD =Yes |
|  |  | 9 | 1246 | 5.5 | 11(15.82) | 0.010(-0.002,0.021) | CHADS2VASC = 0, 1 |
|  | Dabigatran vs Rivaroxaban | 1 | 611 | 4.5 | 8(19.62) | -0.002(-0.02,0.016) | eGFR = 90+ & Region = Midwest, Northeast |
|  |  | 2 | 1551 | 5.7 | 23(23.19) | -0.012(-0.02,-0.003) | eGFR = 90+ & Region = South, West |
|  |  | 3 | 1174 | 6.5 | 11(13.9) | -0.009(-0.017,-0.002) | eGFR = 15-30, 30-45, 45-60, 60-90 & HASBLED = 0, 1 |
|  |  | 4 | 1489 | 5 | 75(81.06) | 0.008(-0.013,0.029) | eGFR = 15-30, 30-45, 45-60, 60-90 & HASBLED = 2, 3, 4, 5, 6, 7, 8 & CAD =Yes & MI =Yes |
|  |  | 5 | 3160 | 6.8 | 109(49.6) | 0.005(-0.007,0.018) | eGFR = 15-30, 30-45, 45-60, 60-90 & HASBLED = 2, 3, 4, 5, 6, 7, 8 & CAD =Yes & MI =No |
|  |  | 6 | 3466 | 6.9 | 80(30.35) | -0.015(-0.022,-0.007) | eGFR = 15-30, 30-45, 45-60, 60-90 & HASBLED = 2, 3, 4, 5, 6, 7, 8 & CAD =No |
|  | Apixaban vs Warfarin | 1 | 383 | 5 | 22(80.68) | -0.020(-0.057,0.017) | Race = Asian, Black, Hispanic & eGFR = 15-30, 30-45, 45-60 & Age < 74 |
|  |  | 2 | 779 | 4.6 | 46(89.29) | -0.054(-0.079,-0.029) | Race = Asian, Black, Hispanic & eGFR = 15-30, 30-45, 45-60 & Age ≥ 74 |
|  |  | 3 | 572 | 6.9 | 19(42.35) | -0.023(-0.046,-0.001) | Race = Asian, Black, Hispanic & eGFR = 60-90, 90+ & HF =No & Age ≥ 71.5 |
|  |  | 4 | 606 | 5.1 | 9(24.1) | -0.005(-0.02,0.011) | Race = Asian, Black, Hispanic & eGFR = 60-90, 90+ & HF =No & Age < 71.5 |
|  |  | 5 | 986 | 5.3 | 38(66.17) | -0.023(-0.042,-0.004) | Race = Asian, Black, Hispanic & eGFR = 60-90, 90+ & HF =Yes |
|  |  | 6 | 3505 | 6 | 113(43.75) | -0.021(-0.031,-0.012) | Race = White, Other & Age ≥ 70.5 & eGFR = 60-90, 90+ & Non.skin.cancer =No |
|  |  | 7 | 1191 | 6.7 | 64(76.47) | -0.025(-0.047,-0.004) | Race = White, Other & Age ≥ 70.5 & eGFR = 60-90, 90+ & Non.skin.cancer =Yes |
|  |  | 8 | 3019 | 6.6 | 148(72.08) | -0.033(-0.045,-0.021) | Race = White, Other & Age ≥ 70.5 & eGFR = 15-30, 30-45, 45-60 & CCB =No |
|  |  | 9 | 1196 | 5.1 | 72(87.92) | -0.021(-0.045,0.002) | Race = White, Other & Age ≥ 70.5 & eGFR = 15-30, 30-45, 45-60 & CCB =Yes |
|  |  | 10 | 3652 | 5.9 | 63(27.45) | -0.015(-0.022,-0.008) | Race = White, Other & Age < 70.5 & Non.skin.cancer =No & Liver.Disease =No |
|  |  | 11 | 672 | 4.4 | 26(68.02) | -0.030(-0.053,-0.007) | Race = White, Other & Age < 70.5 & Non.skin.cancer =No & Liver.Disease =Yes |
|  |  | 12 | 364 | 4 | 13(61.44) | 0.001(-0.034,0.036) | Race = White, Other & Age < 70.5 & Non.skin.cancer =Yes & Obesity =Yes |
|  |  | 13 | 448 | 4.7 | 11(40.17) | -0.019(-0.039,0.002) | Race = White, Other & Age < 70.5 & Non.skin.cancer =Yes & Obesity =No |
|  | Dabigatran vs Warfarin | 1 | 1792 | 6.9 | 85(68.14) | -0.004(-0.025,0.016) | Race = Asian, Black |
|  |  | 2 | 910 | 5.1 | 35(63.78) | -0.018(-0.038,0.002) | Race = Hispanic, White, Other & Prescribed Thiaziades =No & Cardioversion =Yes |
|  |  | 3 | 7284 | 6.8 | 342(69.2) | -0.015(-0.025,-0.004) | Race = Hispanic, White, Other & Prescribed Thiaziades =No & Cardioversion =No & CHADS2VASC = 3+ |
|  |  | 4 | 1176 | 6.9 | 13(17.75) | -0.008(-0.019,0.003) | Race = Hispanic, White, Other & Prescribed Thiaziades =No & Cardioversion =No & CHADS2VASC = 0, 1, 2 |
|  |  | 5 | 2225 | 6.7 | 88(49.19) | -0.004(-0.02,0.012) | Race = Hispanic, White, Other & Prescribed Thiaziades =Yes & Age ≥ 64.5 |
|  |  | 6 | 542 | 6.5 | 14(36.91) | -0.016(-0.035,0.004) | Race = Hispanic, White, Other & Prescribed Thiaziades =Yes & Age < 64.5 |
|  | Rivaroxaban vs Warfarin | 1 | 677 | 4.4 | 52(105.88) | 0.000(-0.037,0.036) | Age ≥ 64.5 & Race = Asian, Black & eGFR = 15-30, 30-45, 45-60 |
|  |  | 2 | 978 | 6.9 | 37(51.55) | 0.006(-0.017,0.029) | Age ≥ 64.5 & Race = Asian, Black & eGFR = 60-90, 90+ |
|  |  | 3 | 5131 | 6.7 | 159(41.57) | -0.003(-0.011,0.005) | Age ≥ 64.5 & Race = Hispanic, White, Other & HASBLED = 0, 1, 2, 3 & CCB =No |
|  |  | 4 | 1354 | 5.5 | 63(60.64) | 0.011(-0.008,0.031) | Age ≥ 64.5 & Race = Hispanic, White, Other & HASBLED = 0, 1, 2, 3 & CCB =Yes |
|  |  | 5 | 2322 | 5.9 | 145(100.61) | -0.016(-0.031,0) | Age ≥ 64.5 & Race = Hispanic, White, Other & HASBLED = 4, 5, 6, 7 & Depression =Yes |
|  |  | 6 | 2529 | 6.5 | 154(89.39) | -0.027(-0.043,-0.011) | Age ≥ 64.5 & Race = Hispanic, White, Other & HASBLED = 4, 5, 6, 7 & Depression =No |
|  |  | 7 | 450 | 3.9 | 22(83.74) | -0.017(-0.05,0.016) | Age < 64.5 & COPD =Yes |
|  |  | 8 | 487 | 4.2 | 10(37.6) | -0.009(-0.028,0.011) | Age < 64.5 & COPD =No & Prescribed Diltiazem =Yes |
|  |  | 9 | 2475 | 5.8 | 52(35.53) | 0.003(-0.007,0.012) | Age < 64.5 & COPD =No & Prescribed Diltiazem =No |
| All-cause Mortality | Apixaban vs Dabigatran | 1 | 553 | 6.3 | 11(24.96) | -0.009(-0.034,0.015) | Age ≥ 59.5 & Age < 63 |
|  |  | 2 | 1012 | 6.5 | 109(141.98) | -0.065(-0.098,-0.032) | Age ≥ 63 & Obs.Sleep.Apnea =No & HF =Yes |
|  |  | 3 | 2168 | 6.9 | 51(30.24) | -0.011(-0.022,0) | Age ≥ 63 & Obs.Sleep.Apnea =No & HF =No |
|  |  | 4 | 871 | 5 | 30(47.91) | -0.031(-0.051,-0.01) | Age ≥ 63 & Obs.Sleep.Apnea =Yes |
|  |  | 5 | 1345 | 6.5 | 12(14.85) | 0.000(-0.01,0.01) | Age < 59.5 |
|  | Apixaban  vs Rivaroxaban | 1 | 335 | 5.2 | 14(56.14) | -0.022(-0.057,0.012) | PCI/CABG =Yes & ACE.ARB =Yes & Smoke =No & Age < 74 |
|  |  | 2 | 352 | 5 | 31(122.1) | -0.080(-0.124,-0.036) | PCI/CABG =Yes & ACE.ARB =Yes & Smoke =No & Age ≥ 74 |
|  |  | 3 | 642 | 4.9 | 33(77.21) | 0.008(-0.021,0.037) | PCI/CABG =Yes & ACE/ARB =Yes & Smoke =Yes |
|  |  | 4 | 376 | 4.2 | 28(121.42) | -0.033(-0.076,0.01) | PCI/CABG =Yes & ACE/ARB =No & Smoke =Yes |
|  |  | 5 | 415 | 3.8 | 17(57.27) | -0.030(-0.06,0) | PCI/CABG =Yes & ACE/ARB =No & Smoke =No |
|  |  | 6 | 446 | 5 | 25(77.99) | -0.029(-0.064,0.007) | PCI/CABG =No & Ischemic Stroke =Yes & Prescribed Statins =No |
|  |  | 7 | 535 | 5.1 | 20(51.15) | -0.009(-0.04,0.021) | PCI/CABG =No & Ischemic Stroke =Yes & Prescribed Statins =Yes & Hypothyroidism =No |
|  |  | 8 | 306 | 4.2 | 16(68.68) | 0.036(-0.01,0.081) | PCI/CABG =No & Ischemic Stroke =Yes & Prescribed Statins =Yes & Hypothyroidism =Yes |
|  |  | 9 | 799 | 4.7 | 17(34.01) | -0.008(-0.024,0.008) | PCI/CABG =No & Ischemic Stroke =No & Age < 68 & Prescribed Antiulcer =Yes |
|  |  | 10 | 3269 | 5.5 | 38(17.93) | -0.001(-0.008,0.005) | PCI/CABG =No & Ischemic Stroke =No & Age < 68 & Prescribed Antiulcer =No |
|  |  | 11 | 4159 | 5.2 | 144(46.54) | -0.003(-0.012,0.007) | PCI/CABG =No & Ischemic Stroke =No & Age ≥ 68 & Non.skin.cancer =No |
|  |  | 12 | 1193 | 5.4 | 64(73.88) | -0.003(-0.025,0.019) | PCI/CABG =No & Ischemic Stroke =No & Age ≥ 68 & Non.skin.cancer =Yes |
|  | Dabigatran vs Rivaroxaban | 1 | 611 | 4.5 | 22(51.44) | 0.014(-0.018,0.046) | eGFR = 90+ & Region = Midwest, Northeast |
|  |  | 2 | 1551 | 5.7 | 26(25.45) | -0.012(-0.021,-0.003) | eGFR = 90+ & Region = South, West |
|  |  | 3 | 3967 | 6.9 | 45(15.68) | 0.003(-0.004,0.009) | eGFR = 15-30, 30-45, 45-60, 60-90 & HF =No & Age < 75 |
|  |  | 4 | 1869 | 6.8 | 79(52.22) | 0.003(-0.014,0.021) | eGFR = 15-30, 30-45, 45-60, 60-90 & HF =No & Age ≥ 77 |
|  |  | 5 | 480 | 6.2 | 15(37.13) | -0.007(-0.029,0.016) | eGFR = 15-30, 30-45, 45-60, 60-90 & HF =No & Age ≥ 75 & Age < 77 |
|  |  | 6 | 531 | 4.8 | 24(70.27) | 0.027(-0.012,0.066) | eGFR = 15-30, 30-45, 45-60, 60-90 & HF =Yes & Obesity =Yes & Statins =No |
|  |  | 7 | 713 | 5 | 59(116.71) | 0.059(0.018,0.1) | eGFR = 15-30, 30-45, 45-60, 60-90 & HF =Yes & Obesity =Yes & Statins =Yes |
|  |  | 8 | 554 | 5.4 | 65(147.72) | 0.104(0.042,0.166) | eGFR = 15-30, 30-45, 45-60, 60-90 & HF =Yes & Obesity =No & MI =Yes |
|  |  | 9 | 1175 | 6.5 | 98(104.66) | 0.017(-0.016,0.051) | eGFR = 15-30, 30-45, 45-60, 60-90 & HF =Yes & Obesity =No & MI =No |
|  | Apixaban vs Warfarin | 1 | 405 | 6.1 | 51(159.88) | -0.082(-0.134,-0.031) | Diabetes =Yes & Race = Asian, Black, Hispanic & Insulin =Yes |
|  |  | 2 | 541 | 5.1 | 51(121.27) | -0.063(-0.1,-0.026) | Diabetes =Yes & Race = Asian, Black, Hispanic & Insulin =No & eGFR = 15-30, 30-45, 45-60 |
|  |  | 3 | 902 | 6.9 | 54(88.78) | -0.033(-0.057,-0.009) | Diabetes =Yes & Race = Asian, Black, Hispanic & Insulin =No & eGFR = 60-90, 90+ |
|  |  | 4 | 2609 | 6.8 | 223(109.57) | -0.059(-0.076,-0.043) | Diabetes =Yes & Race = White, Other & Non.skin.cancer =No & Obesity =No |
|  |  | 5 | 2037 | 5.1 | 112(78.62) | -0.037(-0.053,-0.021) | Diabetes =Yes & Race = White, Other & Non.skin.cancer =No & Obesity =Yes |
|  |  | 6 | 1502 | 6.3 | 135(127.74) | -0.039(-0.064,-0.014) | Diabetes =Yes & Race = White, Other & Non.skin.cancer =Yes |
|  |  | 7 | 828 | 6.7 | 34(46.14) | -0.019(-0.044,0.006) | Diabetes =No & Age ≥ 72 & HF =No & Prescribed Thiaziades =Yes |
|  |  | 8 | 2503 | 6.6 | 98(47.73) | -0.030(-0.043,-0.018) | Diabetes =No & Age ≥ 72 & HF =No & Prescribed Thiaziades =No |
|  |  | 9 | 1930 | 5.8 | 166(118.69) | -0.021(-0.043,0.002) | Diabetes =No & Age ≥ 72 & HF =Yes & Prescribed Digoxin =No |
|  |  | 10 | 373 | 6.3 | 40(147.06) | -0.027(-0.078,0.024) | Diabetes =No & Age ≥ 72 & HF =Yes & Prescribed Digoxin =Yes |
|  |  | 11 | 2594 | 5.5 | 55(32.82) | -0.017(-0.026,-0.008) | Diabetes =No & Age < 72 & Hist.Major.Bleed =No & HASBLED = 0, 1, 2, 3 |
|  |  | 12 | 381 | 4.1 | 22(85.78) | -0.041(-0.082,0) | Diabetes =No & Age < 72 & Hist.Major.Bleed =No & HASBLED = 4, 5, 6, 7 |
|  |  | 13 | 341 | 5.9 | 15(71.14) | -0.024(-0.061,0.014) | Diabetes =No & Age < 72 & Hist.Major.Bleed =Yes & Smoke =Yes |
|  |  | 14 | 427 | 5.6 | 11(35.51) | -0.006(-0.034,0.021) | Diabetes =No & Age < 72 & Hist.Major.Bleed =Yes & Smoke =No |
|  | Dabigatran vs Warfarin | 1 | 1131 | 6.7 | 51(59.32) | -0.011(-0.035,0.014) | VHD =Yes & HF =No & Smoke =Yes |
|  |  | 2 | 2296 | 6.8 | 93(48.22) | -0.031(-0.044,-0.019) | VHD =Yes & HF =No & Smoke =No |
|  |  | 3 | 1815 | 6.5 | 233(166.34) | 0.049(0.006,0.093) | VHD =Yes & HF =Yes & Age ≥ 76.5 |
|  |  | 4 | 1431 | 6.8 | 158(144.37) | -0.004(-0.04,0.032) | VHD =Yes & HF =Yes & Age < 76.5 & Hist.Major.Bleed =No |
|  |  | 5 | 612 | 5.2 | 66(159.98) | 0.082(0.014,0.15) | VHD =Yes & HF =Yes & Age < 76.5 & Hist.Major.Bleed =Yes |
|  |  | 6 | 1216 | 6.9 | 74(70.37) | -0.003(-0.028,0.022) | VHD =No & eGFR = 60-90, 90+ & Age ≥ 71.5 & Prescribed ORC |
|  |  | 7 | 691 | 5.7 | 37(63.5) | 0.001(-0.05,0.051) | VHD =No & eGFR = 60-90, 90+ & Age ≥ 71.5 & Prescribed ORC = No |
|  |  | 8 | 899 | 5.7 | 27(49.91) | -0.004(-0.034,0.027) | VHD =No & eGFR = 60-90, 90+ & Age < 71.5 & eGFR = 90+ |
|  |  | 9 | 1658 | 6.5 | 40(33.85) | 0.000(-0.016,0.016) | VHD =No & eGFR = 60-90, 90+ & Age < 71.5 & eGFR = 60-90 |
|  |  | 10 | 735 | 6.6 | 66(108.85) | -0.044(-0.077,-0.012) | VHD =No & eGFR = 15-30, 30-45, 45-60 & Not prescribed ORC |
|  |  | 11 | 1445 | 6.9 | 147(121.85) | 0.011(-0.02,0.042) | VHD =No & eGFR = 15-30, 30-45, 45-60 & Prescribed ORC= Yes |
|  | Rivaroxaban vs Warfarin | 1 | 908 | 6.7 | 72(93.48) | -0.058(-0.084,-0.032) | Region = Midwest & Hypothyroidism =Yes |
|  |  | 2 | 870 | 6.3 | 71(105.12) | -0.017(-0.049,0.014) | Region = Midwest & Hypothyroidism =No & Smoke =Yes |
|  |  | 3 | 605 | 6.5 | 36(72.48) | -0.004(-0.038,0.031) | Region = Midwest & Hypothyroidism =No & Smoke =No & CAD =Yes |
|  |  | 4 | 607 | 5.4 | 26(52.66) | -0.019(-0.045,0.007) | Region = Midwest & Hypothyroidism =No & Smoke =No & CAD =No |
|  |  | 5 | 6019 | 6.9 | 431(89.01) | -0.035(-0.046,-0.025) | Region = Northeast, South, West & Prescribed Antiplatelet =No & Age ≥ 70 & Age < 84 |
|  |  | 6 | 1375 | 4.7 | 125(124.5) | 0.010(-0.02,0.04) | Region = Northeast, South, West & Antiplatelet =No & Age ≥ 84 |
|  |  | 7 | 663 | 6.8 | 67(153.13) | -0.034(-0.073,0.005) | Region = Northeast, South, West & Prescribed Antiplatelet =No & Age < 70 & Systolic HF =Yes |
|  |  | 8 | 3549 | 5.9 | 96(40.85) | -0.009(-0.018,0) | Region = Northeast, South, West & Prescribed Antiplatelet =No & Age < 70 & Systolic HF =No |
|  |  | 9 | 765 | 5.9 | 88(156.84) | -0.035(-0.071,0.002) | Region = Northeast, South, West & Prescribed Antiplatelet =Yes & eGFR = 15-30, 30-45, 45-60 |
|  |  | 10 | 1042 | 6.3 | 68(82.91) | -0.014(-0.04,0.012) | Region = Northeast, South, West & Prescribed Antiplatelet =Yes & eGFR = 60-90, 90+ |

Table 6: Apixaban vs. Dabigatran: Baseline characteristics pre-and post-matching.

|  | **Before Matching** | | | | **After Matching** | | | |
| --- | --- | --- | --- | --- | --- | --- | --- | --- |
|  | **Apixaban** | **Dabigatran** | **p-value** | **SMD** | **Apixaban** | **Dabigatran** | **p-value** | **SMD** |
| n | 11350 | 3435 |  |  | 2514 | 3435 |  |  |
| Age | 72.30 (10.52) | 67.24 (11.09) |  | 0.468 | 68.42 (10.61) | 67.24 (11.09) |  | 0.109 |
| eGFR groups |  |  | <0.001 | 0.254 |  |  | 0.523 | 0.046 |
| 15-30 | 327 ( 2.9) | 39 ( 1.1) |  |  | 42 ( 1.7) | 39 ( 1.1) |  |  |
| 30-45 | 1213 (10.7) | 222 ( 6.5) |  |  | 167 ( 6.6) | 222 ( 6.5) |  |  |
| 45-60 | 2278 (20.1) | 594 ( 17.3) |  |  | 433 (17.2) | 594 ( 17.3) |  |  |
| 60-90 | 5916 (52.1) | 1877 ( 54.6) |  |  | 1363 (54.2) | 1877 ( 54.6) |  |  |
| 90+ | 1616 (14.2) | 703 ( 20.5) |  |  | 509 (20.2) | 703 ( 20.5) |  |  |
| CHADS2VASC |  |  | <0.001 | 0.305 |  |  | 0.023 | 0.081 |
| 0 | 139 ( 1.2) | 106 ( 3.1) |  |  | 70 ( 2.8) | 106 ( 3.1) |  |  |
| 1 | 548 ( 4.8) | 330 ( 9.6) |  |  | 206 ( 8.2) | 330 ( 9.6) |  |  |
| 2 | 1131 (10.0) | 532 ( 15.5) |  |  | 343 (13.6) | 532 ( 15.5) |  |  |
| 3 | 9532 (84.0) | 2467 ( 71.8) |  |  | 1895 (75.4) | 2467 ( 71.8) |  |  |
| HASBLED |  |  | <0.001 | 0.355 |  |  | 0.029 | 0.109 |
| 0 | 196 ( 1.7) | 128 ( 3.7) |  |  | 81 ( 3.2) | 128 ( 3.7) |  |  |
| 1 | 999 ( 8.8) | 558 ( 16.2) |  |  | 348 (13.8) | 558 ( 16.2) |  |  |
| 2 | 2685 (23.7) | 954 ( 27.8) |  |  | 675 (26.8) | 954 ( 27.8) |  |  |
| 3 | 3382 (29.8) | 974 ( 28.4) |  |  | 725 (28.8) | 974 ( 28.4) |  |  |
| 4 | 2576 (22.7) | 547 ( 15.9) |  |  | 467 (18.6) | 547 ( 15.9) |  |  |
| 5 | 1147 (10.1) | 230 ( 6.7) |  |  | 178 ( 7.1) | 230 ( 6.7) |  |  |
| 6 | 327 ( 2.9) | 35 ( 1.0) |  |  | 36 ( 1.4) | 35 ( 1.0) |  |  |
| 7 | 36 ( 0.3) | 7 ( 0.2) |  |  | 4 ( 0.2) | 7 ( 0.2) |  |  |
| 8 | NA | NA |  |  | NA | NA |  |  |
| Region |  |  | <0.001 | 0.124 |  |  | 0.187 | 0.057 |
| Midwest | 1922 (16.9) | 439 ( 12.8) |  |  | 363 (14.4) | 439 ( 12.8) |  |  |
| Northeast | 1912 (16.8) | 558 ( 16.2) |  |  | 387 (15.4) | 558 ( 16.2) |  |  |
| South | 6498 (57.3) | 2091 ( 60.9) |  |  | 1493 (59.4) | 2091 ( 60.9) |  |  |
| West | 1018 ( 9.0) | 347 ( 10.1) |  |  | 271 (10.8) | 347 ( 10.1) |  |  |
| Race |  |  | <0.001 | 0.163 |  |  | <0.001 | 0.139 |
| Asian | 261 ( 2.3) | 96 ( 2.8) |  |  | 51 ( 2.0) | 96 ( 2.8) |  |  |
| Black | 1246 (11.0) | 272 ( 7.9) |  |  | 242 ( 9.6) | 272 ( 7.9) |  |  |
| Hispanic | 714 ( 6.3) | 212 ( 6.2) |  |  | 140 ( 5.6) | 212 ( 6.2) |  |  |
| White | 7987 (70.4) | 2614 ( 76.1) |  |  | 1827 (72.7) | 2614 ( 76.1) |  |  |
| Other | 1142 (10.1) | 241 ( 7.0) |  |  | 254 (10.1) | 241 ( 7.0) |  |  |
| Gender:female | 5376 (47.4) | 1251 ( 36.4) | <0.001 | 0.223 | 989 (39.3) | 1251 ( 36.4) | 0.023 | 0.06 |
| **Comorbidities/Conditions/Procedures** | | | | | | | | |
| HF | 4227 (37.2) | 1010 ( 29.4) | <0.001 | 0.167 | 773 (30.7) | 1010 ( 29.4) | 0.276 | 0.029 |
| HTN | 10560 (93.0) | 3104 ( 90.4) | <0.001 | 0.097 | 2291 (91.1) | 3104 ( 90.4) | 0.338 | 0.026 |
| Thrombo | 2562 (22.6) | 623 ( 18.1) | <0.001 | 0.11 | 468 (18.6) | 623 ( 18.1) | 0.662 | 0.012 |
| Diabetes | 4747 (41.8) | 1346 ( 39.2) | 0.006 | 0.054 | 1007 (40.1) | 1346 ( 39.2) | 0.515 | 0.018 |
| CAD | 6312 (55.6) | 1822 ( 53.0) | 0.008 | 0.052 | 1324 (52.7) | 1822 ( 53.0) | 0.794 | 0.008 |
| PAD | 1640 (14.4) | 396 ( 11.5) | <0.001 | 0.087 | 284 (11.3) | 396 ( 11.5) | 0.813 | 0.007 |
| HMB | 2773 (24.4) | 804 ( 23.4) | 0.227 | 0.024 | 574 (22.8) | 804 ( 23.4) | 0.626 | 0.014 |
| HIB | 256 ( 2.3) | 66 ( 1.9) | 0.268 | 0.023 | 48 ( 1.9) | 66 ( 1.9) | 1 | 0.001 |
| Liver Disease | 1874 (16.5) | 522 ( 15.2) | 0.071 | 0.036 | 391 (15.6) | 522 ( 15.2) | 0.734 | 0.01 |
| Alcoholism | 623 ( 5.5) | 184 ( 5.4) | 0.798 | 0.006 | 142 ( 5.6) | 184 ( 5.4) | 0.667 | 0.013 |
| Obesity | 4214 (37.1) | 1125 ( 32.8) | <0.001 | 0.092 | 881 (35.0) | 1125 ( 32.8) | 0.069 | 0.048 |
| Smoke | 4208 (37.1) | 1058 ( 30.8) | <0.001 | 0.133 | 917 (36.5) | 1058 ( 30.8) | <0.001 | 0.12 |
| Falls | 1955 (17.2) | 362 ( 10.5) | <0.001 | 0.194 | 340 (13.5) | 362 ( 10.5) | <0.001 | 0.092 |
| AKI | 2186 (19.3) | 365 ( 10.6) | <0.001 | 0.244 | 312 (12.4) | 365 ( 10.6) | 0.036 | 0.056 |
| VHD | 5700 (50.2) | 1711 ( 49.8) | 0.688 | 0.008 | 1165 (46.3) | 1711 ( 49.8) | 0.009 | 0.069 |
| Non skin cancer | 2344 (20.7) | 618 ( 18.0) | 0.001 | 0.067 | 446 (17.7) | 618 ( 18.0) | 0.83 | 0.007 |
| Recent major bleed | 60 ( 0.5) | 21 ( 0.6) | 0.657 | 0.011 | 7 ( 0.3) | 21 ( 0.6) | 0.097 | 0.05 |
| Recent Thrombo | 598 ( 5.3) | 134 ( 3.9) | 0.001 | 0.065 | 119 ( 4.7) | 134 ( 3.9) | 0.132 | 0.041 |
| Hyperlipidemia | 10113 (89.1) | 3074 ( 89.5) | 0.54 | 0.013 | 2271 (90.3) | 3074 ( 89.5) | 0.307 | 0.028 |
| Ischemic Stroke | 1837 (16.2) | 412 ( 12.0) | <0.001 | 0.121 | 335 (13.3) | 412 ( 12.0) | 0.136 | 0.04 |
| MI | 2115 (18.6) | 519 ( 15.1) | <0.001 | 0.094 | 409 (16.3) | 519 ( 15.1) | 0.237 | 0.032 |
| COPD | 1755 (15.5) | 456 ( 13.3) | 0.002 | 0.062 | 337 (13.4) | 456 ( 13.3) | 0.915 | 0.004 |
| Obs.Sleep.Apnea | 2690 (23.7) | 853 ( 24.8) | 0.181 | 0.026 | 657 (26.1) | 853 ( 24.8) | 0.267 | 0.03 |
| Systolic HF | 1850 (16.3) | 349 ( 10.2) | <0.001 | 0.182 | 298 (11.9) | 349 ( 10.2) | 0.042 | 0.054 |
| Cardioversion | 1530 (13.5) | 505 ( 14.7) | 0.073 | 0.035 | 351 (14.0) | 505 ( 14.7) | 0.444 | 0.021 |
| Ablation | 313 ( 2.8) | 162 ( 4.7) | <0.001 | 0.103 | 87 ( 3.5) | 162 ( 4.7) | 0.02 | 0.063 |
| Pacemaker/ICD | 1581 (13.9) | 384 ( 11.2) | <0.001 | 0.083 | 283 (11.3) | 384 ( 11.2) | 0.958 | 0.002 |
| PCI/CABG | 2081 (18.3) | 571 ( 16.6) | 0.023 | 0.045 | 442 (17.6) | 571 ( 16.6) | 0.349 | 0.025 |
| Depression | 4550 (40.1) | 1203 ( 35.0) | <0.001 | 0.105 | 992 (39.5) | 1203 ( 35.0) | 0.001 | 0.092 |
| Dementia | 939 ( 8.3) | 154 ( 4.5) | <0.001 | 0.156 | 161 ( 6.4) | 154 ( 4.5) | 0.001 | 0.085 |
| Hypothyroidism | 3863 (34.0) | 1095 ( 31.9) | 0.02 | 0.046 | 770 (30.6) | 1095 ( 31.9) | 0.318 | 0.027 |
| Thyrotoxicosis | 586 ( 5.2) | 212 ( 6.2) | 0.024 | 0.044 | 129 ( 5.1) | 212 ( 6.2) | 0.099 | 0.045 |
| Ulcer | 697 ( 6.1) | 204 ( 5.9) | 0.694 | 0.008 | 141 ( 5.6) | 204 ( 5.9) | 0.63 | 0.014 |
| **Medications** | | | | | | | | |
| Antiplatelet | 1386 (12.2) | 364 ( 10.6) | 0.011 | 0.051 | 273 (10.9) | 364 ( 10.6) | 0.779 | 0.008 |
| NSAIDS | 1376 (12.1) | 444 ( 12.9) | 0.221 | 0.024 | 291 (11.6) | 444 ( 12.9) | 0.128 | 0.041 |
| Amiodarone | 1367 (12.0) | 364 ( 10.6) | 0.023 | 0.046 | 299 (11.9) | 364 ( 10.6) | 0.126 | 0.041 |
| Dronedarone | 296 ( 2.6) | 169 ( 4.9) | <0.001 | 0.122 | 84 ( 3.3) | 169 ( 4.9) | 0.004 | 0.079 |
| OAAD | 1264 (11.1) | 484 ( 14.1) | <0.001 | 0.089 | 324 (12.9) | 484 ( 14.1) | 0.194 | 0.035 |
| Digoxin | 821 ( 7.2) | 371 ( 10.8) | <0.001 | 0.125 | 242 ( 9.6) | 371 ( 10.8) | 0.153 | 0.039 |
| Diltiazem | 2064 (18.2) | 626 ( 18.2) | 0.979 | 0.001 | 453 (18.0) | 626 ( 18.2) | 0.866 | 0.005 |
| Verapamil | 161 ( 1.4) | 68 ( 2.0) | 0.024 | 0.043 | 38 ( 1.5) | 68 ( 2.0) | 0.212 | 0.036 |
| ORC | 7630 (67.2) | 2171 ( 63.2) | <0.001 | 0.085 | 1663 (66.1) | 2171 ( 63.2) | 0.02 | 0.062 |
| ACE/ARB | 6201 (54.6) | 1816 ( 52.9) | 0.072 | 0.035 | 1341 (53.3) | 1816 ( 52.9) | 0.737 | 0.009 |
| CCB | 2704 (23.8) | 721 ( 21.0) | 0.001 | 0.068 | 542 (21.6) | 721 ( 21.0) | 0.618 | 0.014 |
| Obeta | 608 ( 5.4) | 184 ( 5.4) | 1 | <0.001 | 115 ( 4.6) | 184 ( 5.4) | 0.192 | 0.036 |
| Loop Diuretics | 2831 (24.9) | 649 ( 18.9) | <0.001 | 0.147 | 502 (20.0) | 649 ( 18.9) | 0.316 | 0.027 |
| Thiaziades | 2692 (23.7) | 858 ( 25.0) | 0.136 | 0.029 | 551 (21.9) | 858 ( 25.0) | 0.007 | 0.072 |
| Statins | 6089 (53.6) | 1704 ( 49.6) | <0.001 | 0.081 | 1323 (52.6) | 1704 ( 49.6) | 0.023 | 0.06 |
| Insulin | 894 ( 7.9) | 233 ( 6.8) | 0.038 | 0.042 | 194 ( 7.7) | 233 ( 6.8) | 0.184 | 0.036 |
| Metformin | 1731 (15.3) | 523 ( 15.2) | 0.993 | 0.001 | 410 (16.3) | 523 ( 15.2) | 0.272 | 0.03 |
| Odiab | 1468 (12.9) | 426 ( 12.4) | 0.43 | 0.016 | 320 (12.7) | 426 ( 12.4) | 0.736 | 0.01 |
| Antiulcer agents | 3189 (28.1) | 774 ( 22.5) | <0.001 | 0.128 | 659 (26.2) | 774 ( 22.5) | 0.001 | 0.086 |
| Unless otherwise noted, data are presented as n (%) for categorical variables and mean (SD) for continuous variables. CABG indicates coronary artery bypass graft; COPD, chronic obstructive pulmonary disease; eGFR, glomerular filtration rate; GI, gastrointestinal; ICD, implantable cardioverter-defibrillator; NSAID, nonsteroidal anti-inflammatory drug; PCI, percutaneous coronary intervention; SMD, standardized mean difference. NA indicate counts that are less than 10, and we masked those cells in accordance with OptumLabs® data use policy.  *The CHADS2VASC score ranges from 0 to 9; higher score indicates higher risk of stroke. A point score is calculated as 1 point each for heart failure, hypertension, diabetes mellitus, vascular disease, age 65 to 74 years, and female sex; 2 points for age ≥75 y and prior stroke, TIA, or thromboembolism.  †The HASBLED score ranges from 0 to 9; higher score indicates higher risk of bleeding. A point score is calculated as 1 point each for hypertension, abnormal kidney function, abnormal liver function, prior stroke, prior bleeding or bleeding predisposition, labile international normalized ratio (INR), older than 65 years, medication usage predisposing to bleeding, and alcohol use. This study did not consider INR, so the range of HASBLED was 0–8. | | | | | | | | |

References

1. Wallace PJ, Shah ND, Dennen T, Bleicher PA, Crown WH. Optum Labs: building a novel node in the learning health care system. *Health Affairs.* 2014;33(7):1187-1194.

2. Yao X, Inselman JW, Ross JS, et al. Comparative effectiveness and safety of oral anticoagulants across kidney function in patients with atrial fibrillation. *Circulation: Cardiovascular Quality and Outcomes.* 2020;13(10):e006515.

3. Fanola CL, Giugliano RP, Ruff CT, et al. A novel risk prediction score in atrial fibrillation for a net clinical outcome from the ENGAGE AF-TIMI 48 randomized clinical trial. *European Heart Journal.* 2017;38(12):888-896.

4. Wilchesky M, Tamblyn RM, Huang A. Validation of diagnostic codes within medical services claims. *Journal of clinical epidemiology.* 2004;57(2):131-141.

5. Tirschwell DL, Longstreth Jr W. Validating administrative data in stroke research. *Stroke.* 2002;33(10):2465-2470.

6. Cunningham A, Stein CM, Chung CP, Daugherty JR, Smalley WE, Ray WA. An automated database case definition for serious bleeding related to oral anticoagulant use. *Pharmacoepidemiology and drug safety.* 2011;20(6):560-566.

7. Arnason T, Wells P, Van Walraven C, Forster A. Accuracy of coding for possible warfarin complications in hospital discharge abstracts. *Thrombosis research.* 2006;118(2):253-262.

8. Schulman S, Kearon C, Scientific SoCoAot, Thrombosis SCotISo, Haemostasis. Definition of major bleeding in clinical investigations of antihemostatic medicinal products in non‐surgical patients. *Journal of thrombosis and haemostasis.* 2005;3(4):692-694.

9. Jasuja GK, Reisman JI, Miller DR, et al. Identifying major hemorrhage with automated data: results of the Veterans Affairs study to improve anticoagulation (VARIA). *Thrombosis research.* 2013;131(1):31-36.

10. Breiman L. Random forests. *Machine learning.* 2001;45(1):5-32.

11. Kursa MB, Rudnicki WR. Feature selection with the Boruta package. *J Stat Softw.* 2010;36(11):1-13.

12. Athey S, Imbens G. Recursive partitioning for heterogeneous causal effects. *Proceedings of the National Academy of Sciences.* 2016;113(27):7353-7360.

13. Zhang W, Le TD, Liu L, Zhou Z-H, Li J. Mining heterogeneous causal effects for personalized cancer treatment. *Bioinformatics.* 2017;33(15):2372-2378.

14. Van Der Laan MJ, Rubin D. Targeted maximum likelihood learning. *The International Journal of Biostatistics.* 2006;2(1).

15. Ngufor C, Warner MA, Murphree DH, et al. Identification of Clinically Meaningful Plasma Transfusion Subgroups Using Unsupervised Random Forest Clustering. Paper presented at: AMIA Annual Symposium Proceedings2017.

16. Van der Laan MJ, Rose S. *Targeted learning: causal inference for observational and experimental data.* Springer Science & Business Media; 2011.

17. Ishwaran H, Kogalur UB, Blackstone EH, Lauer MS. Random survival forests. *The annals of applied statistics.* 2008;2(3):841-860.
